# Adaptation to simulated microgravity in *Streptococcus mutans*

**DOI:** 10.1038/s41526-022-00205-8

**Published:** 2022-06-02

**Authors:** Mizpha C. Fernander, Paris K. Parsons, Billal Khaled, Amina Bradley, Joseph L. Graves, Misty D. Thomas

**Affiliations:** Department of Biology North Carolina Agricultural and Technical State University, Greensboro, NC USA

**Keywords:** Evolution, Microbiology, Infection

## Abstract

Long-term space missions have shown an increased incidence of oral disease in astronauts’ and as a result, are one of the top conditions predicted to impact future missions. Here we set out to evaluate the adaptive response of *Streptococcus mutans* (etiological agent of dental caries) to simulated microgravity. This organism has been well studied on earth and treatment strategies are more predictable. Despite this, we are unsure how the bacterium will respond to the environmental stressors in space. We used experimental evolution for 100-days in high aspect ratio vessels followed by whole genome resequencing to evaluate this adaptive response. Our data shows that planktonic *S. mutans* did evolve variants in three genes (*pknB*, SMU_399 and SMU_1307c) that can be uniquely attributed to simulated microgravity populations. In addition, collection of data at multiple time points showed mutations in three additional genes (SMU_399, *ptsH* and *rex*) that were detected earlier in simulated microgravity populations than in the normal gravity controls, many of which are consistent with other studies. Comparison of virulence-related phenotypes between biological replicates from simulated microgravity and control orientation cultures generally showed few changes in antibiotic susceptibility, while acid tolerance and adhesion varied significantly between biological replicates and decreased as compared to the ancestral populations. Most importantly, our data shows the importance of a parallel normal gravity control, sequencing at multiple time points and the use of biological replicates for appropriate analysis of adaptation in simulated microgravity.

## Introduction

As NASA’s desire to explore and set up human habitation on planets like Mars increases, preventing and/or predicting potential threats to mission objectives are a main priority. Humans face many challenges in space^1,2^; one example being the many microbial challenges they encounter, potentially hindering their own level of performance, and the integrity of their spacecraft and habitat^3,4^. Microbes are not only ubiquitous on earth, but are also readily found on structures inhabited by humans in the spaceflight environment^3,5^. The crew’s normal flora harbors large quantities of microbes making them the most important source of bacterial contamination^6–9^. The human microbiome is essential for our survival as it helps break down food, protects us from pathogens and even primes our immune system. Under conditions of distress, the composition of the normal flora is often altered, ultimately leading to dysbiosis and disease^10–14^. This dysbiosis could be further enhanced by the immune dysregulation encountered during space travel^15–20^. Therefore, infection resulting from opportunistic pathogens is of concern during space flight^21^.

Low-shear stress and reduced gravity can promote microbial dysbiosis and change bacterial physiology^4,22–24^. Experiments have shown that under simulated microgravity, microbes can evolve novel resistance^25^, show enhanced growth^26^, biofilm formation^27,28^, extracellular polysaccharide production^29^, increase production of secondary metabolites^30^ while also showing alterations in pathogenic stress response and virulence^22,31–33^. To reduce incidence of infection in space, both the spacecraft and the crew undergo in-depth microbial screening prior to flight^34^, but this mitigation strategy may not prevent novel evolutionary phenotypes arising within the crew’s microflora that will adapt due to their exposure to the novel environment of space.

Long-term space missions and increased exposure to simulated microgravity and radiation have shown an increased incidence of oral disease in astronauts^35^. As a result, The Space Medicine Exploration Medical Condition List indicates that diagnostic and treatment capabilities for basic dental procedures be made available^36^. In addition, the Integrated Mathematical Medical Model predicts that dental emergencies will be one of the top conditions impacting future mission objectives^37^. Approximately 20% of oral bacteria are streptococci, these organisms are responsible for both early establishment of dental plaque and dental decay^38,39^. The phenotypes of these organisms as they exist on earth are well studied and treatment strategies are more predictable, but these may not hold true during long-term space travel.

The human mouth is a very complex community made up of over 1000 different species and is the second most complex after the GI tract^40,41^. These communities reach cell densities as high as 10^11^ CFU mL^−1^^42^ despite having to endure a constant change in environmental stressors including food intake, temperature, pH and salivary flow^43–45^. The oral microbiome plays a role in not only maintaining oral health, but also in maintaining systemic health as all surfaces of the oral cavity (teeth, gums, tongue etc) are inhabited by microbes which aids in preventing colonization by pathogens^46,47^. Despite this, dental decay remains one of the highest prevailing diseases in humans^48^. Of the oral microbiome residents, *Streptococcus mutans* has been actively studied for its cariogenic properties, as this organism not only causes dental decay, but resides as a member of normal human plaque^49^.

*S. mutans* is a gram-positive facultative anaerobe (Firmicutes) that normally exists as a member of the mature dental biofilm community, but under certain conditions, can become the dominant species leading to dental caries^50,51^. The formation of dental caries is reliant on two factors, 1) an ecological shift that favors the growth of acid producing bacteria and 2) the presence of sucrose in the environment for both fermentation and production of glucans that facilitate attachment of the organism to the tooth, leading to formation of the plaque biofilm which is tolerant to low pH^52^.

Two short-term studies assessed the impact of simulated microgravity on *S. mutans*. Orsini et al.^53^, demonstrated using a High Aspect Ratio Vessel (HARV) for 48 h that *S. mutans* underwent both transcriptomic and metabolomic changes in carbohydrate metabolism and increased stress in the form of hydrogen peroxide susceptibility and noted variation between biological replicates. Orsini et al.^53^ also showed an increase in cellular aggregates, indicative of an increase in cell-to-cell adhesion and/or biofilm formation. Subsequently, Cheng et al.^54^ found that *S. mutans* displayed little changes in growth and hydrogen peroxide tolerance while exhibiting an increase in acid tolerance in response to simulated microgravity. It is hard to determine the value of these short-term studies (48 h) as a typical mission can last 4–6 months at a time. It is highly likely that these short-term studies only represent potential physiological acclimation, as opposed to evolutionary adaptation^55^.

Currently, we have limited knowledge of microbial adaptation in response to long duration space flight. The common human resident: *Streptococcus mutans*, will be taken by every astronaut into space^49^. Therefore, studies such as the present one are essential for predicting which organisms have the potential to evolve into strains with increased virulence and pose a greater risk to human health once in space.

Experimental evolution followed by whole genome resequencing (EERseq) experiments are commonly used for evaluating the genomic changes associated with selection regimes^56–61^. Tirumalai et al.^62^ adapted *E. coli* in simulated microgravity for 1000 generations (~50 days) using HARVs and showed it acquired genomic changes in genes involved in outer membrane protein folding, ion transport and in the stress response. In a similar experiment, they also evaluated the consequences of periodic exposure to antibiotic therapy^63^. To date, these are the only long-term microbial adaptation studies in simulated microgravity in the literature^62,63^. Unfortunately, likely due to the necessity of specialty equipment, adaptation experiments to simulated microgravity are often under-powered with few biological replicates (populations evolved in parallel to capture random variation) and often lacking normal gravity populations for comparison. The absence of a normal gravity control means that Tirumaliai et al.^62,63^ could not legitimately claim that the variants that arose within their simulated microgravity treatment were not just adaptations to some other aspect of their environment, such as the medium.

In the present study, we used HARVs to evaluate the adaptive response of four biological replicates of *S. mutans* to simulated microgravity^64^ over 100-days (~1400 generations) to better understand the consequences of long-term space travel on organisms that reside as normal residents of the host microbiome. The adaptive response was evaluated by performing whole-genome resequencing every three weeks and phenotypes correlated with virulence were assessed after 100-days of adaptation. All adapted populations were compared to the ancestral population as well as to their normal gravity counterparts which differ only by the axis of rotation of the vessel itself^4,65^. Understanding the long-term evolution of the human microbiome in outer space will therefore be an important step in further understanding the effects of space travel on humans and their resident microbes.

## Methods

### Culture strains

*Streptococcus mutans* Clarke strain NCTC 10449 was purchased from the ATCC [https://www.atcc.org/products/25175]. All standard growth experiments were conducted in Brain Heart Infusion (BHI) broth (or agar) at 37 °C with 5% CO_2_ unless otherwise noted.

### Preparation of HARV vessels

High Aspect Rotating Vessels (HARVs) were purchased from Synthecon Inc., Houston, TX and used to culture *S. mutans* under simulated microgravity. Prior to inoculation, the HARVs were cleaned using a mild dish soap and water and rinsed in distilled water twice. All the components were then soaked in a 25% bleach solution for 15 min, rinsed extensively in tap water and then rinsed in distilled water. The HARVs were then loosely reassembled (as per the manufacturer), wrapped in aluminum foil and autoclaved at 121 °C for 20 min. HARVs were then left to cool for 2 h. After they cooled, HARVs were loaded with 5 mL of BHI broth and placed onto the rotator backplate at 25 rpm for 24 h, at 37 °C and 5% CO_2_ to ensure that the HARVs were sterile. This procedure was repeated anytime that contamination was detected over the course of the 100-day evolution experiment.

### Experimental evolution (EE) of *S. mutans* over 100-days of LSMMG exposure

**Fig. 1:**
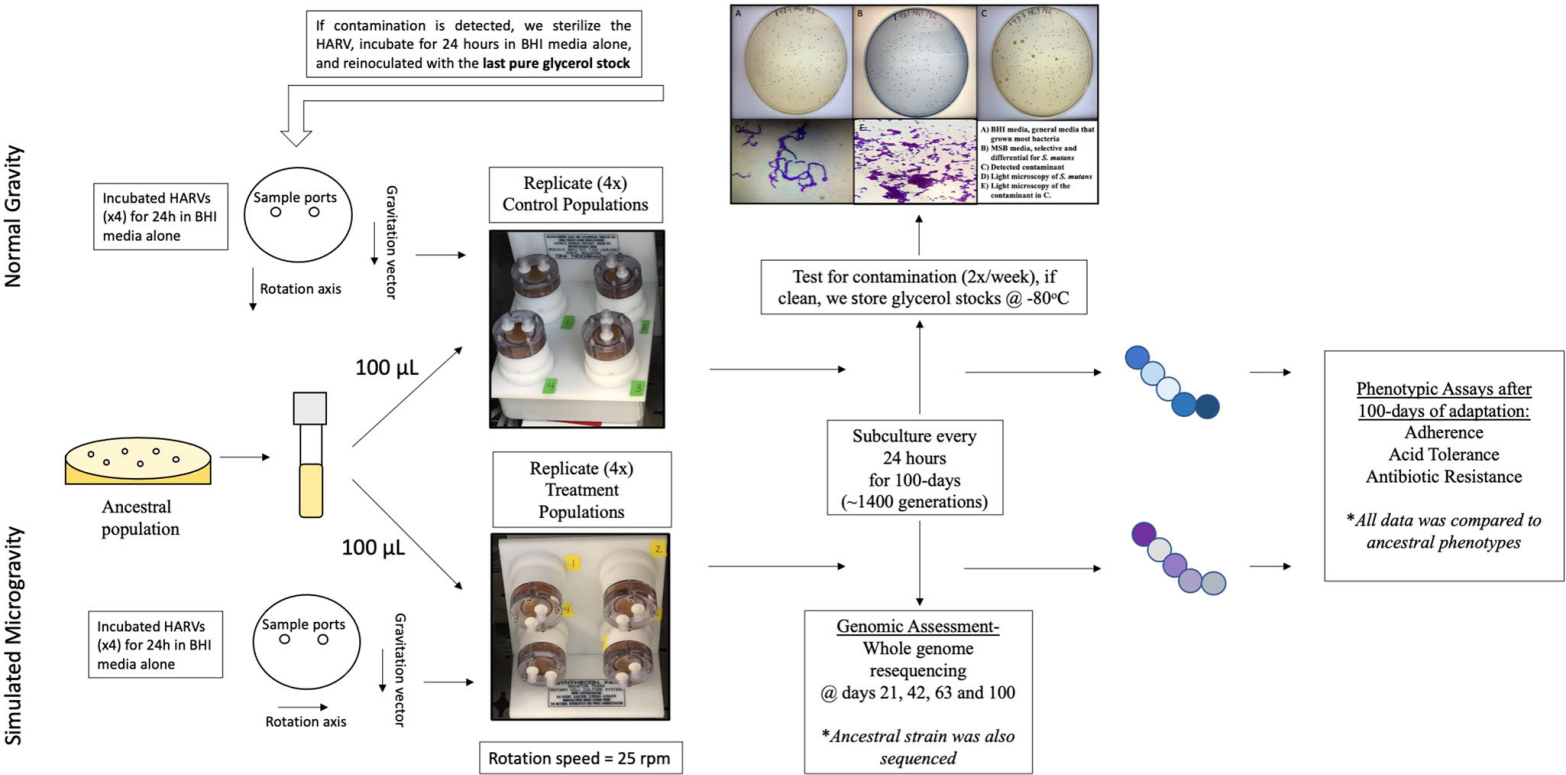
Experimental methods. Schematic representation of the experimental evolution workflow used to adapt *Streptococcus mutans* to simulated microgravity.

The physical and mechanical unloading by simulated microgravity in ground-based systems have been conducted in many studies, characterizing how simulated microgravity impacts various organisms and biological systems^4,6,15,22–27,29,53,54,62,66–73^. An overview of the experimental methods is depicted in Fig. 1. *S. mutans* Clarke strain NCTC 10449 was used to inoculate 3 mL of fresh BHI broth and incubated overnight with shaking at 250 rpm. This stock was then used to streak a BHI agar plate and left to incubate overnight. A single colony was then used to make a glycerol stock deemed the ancestral population. Initial growth curves of the ancestral population in the HARVs showed saturation after ~24 h and the generation time was determined to be ~14 per 24 h (one generation per ~105 min). To begin the EE protocol the ancestral stock was used to start another overnight culture. 100 μL of this overnight culture was then used to inoculate 100 mL of fresh BHI broth, 10 mL of the sub-culture was then loaded into a 10 mL sterile syringe and screwed into one of the two openings on the HARV (inlet), a second empty syringe was placed on the second opening (outlet). 10 mL was then pushed into the inlet while the syringe on the outlet collected the media from the HARV. This was repeated for all 8 HARVs. Four of the HARVs were then incubated on the vertical axis of rotation perpendicular to gravity and deemed normal gravity to serve as the controls and the other four were incubated on the horizontal axis to simulate simulated microgravity. All eight HARVs were incubated at 37 °C overnight with 5% CO_2_ at 25 rpm. After 24 h of growth (~14 generations, Supplementary Fig. 3), the HARVs were then subcultured by adding 10 mL of fresh BHI into the inlet port which pushed the culture from the HARV into an empty syringe attached to the outlet port and returned to the incubator for a new 24 h cycle. This was carried out daily for 100-days (~1400 generations). During the EE study, the HARVs were checked daily for contamination by first, measuring the O.D._600_, values greater than 1 were often indicative of contamination, second, we performed a simple stain with crystal violet on an aliquot of the culture and observed it under a compound light microscope for general shape and arrangement and anything that was uncharacteristic of *S. mutans*. Then twice a week we made glycerol stocks and plated serial dilutions onto both BHI agar and Mitis Salivarius Bacitracin agar (MSB) which is both selective and differential for *S. mutans*. These plates were used to validate the integrity of the glycerol stock which would be used in case of future contamination. Every milestone time point (21-, 42-, 63- and 100-days), the remainder of the culture were pelleted and stored at −80 °C for DNA sequencing. If contamination was detected we would sterilize the HARVs as previously described, inoculate with fresh media for 24 h and then inoculate with the most recently validated glycerol stock.

### Etest® analysis for measuring antibiotic susceptibility

All eight 100-day populations, in addition to the ancestral, were used to swab BHI plates in triplicate. On each of those plates, one Etest® strip (bioMériux) was placed in the center of the plate and left to incubate for 48 h to obtain confluent lawns. Antibiotic susceptibility was then measured by determining the minimum concentration on the strip at which growth was observed. This value was then compared for each of the eight evolved populations against the values obtained for the ancestral population by performing a one-way ANOVA in GraphPad Prism using the multiple comparisons function with the ancestral. In total, six antibiotics were tested including: amoxicillin, penicillin, clindamycin, erythromycin, methicillin, and vancomycin.

### Acid tolerance assays

Acid resistance is one of the main virulence traits in *S. mutans*, we therefore assessed our evolved populations for changes in their ability to survive at low pH. Acid tolerance assays were performed as described elsewhere^74^. In short, after removal of the cultures from the HARVs, a sample of each population was taken and diluted to an O.D._600_ of 0.3. These cultures were then centrifuged to pellet the cells and then washed using 0.2 M glycine pH 6.8. Samples were again pelleted and exposed to 0.2 M glycine pH 2.8 for 0, 20, 30 and 45 min. At the end of the incubation time, samples were pelleted and resuspended in 0.2 M glycine pH 6.8. Serial dilutions of each time point were then plated on BHI agar and CFUs were counted after 24 h of incubation and compared to CFU counts of the ancestral population. Each dilution was plated in triplicate to acquire accurate CFU counts.

### Adhesion assays

The ability to adhere to the tooth pellicle is another main virulence factor required by *S. mutans* to establish itself in a biofilm. Therefore, at 100-days, all eight subcultured populations were assessed for changes in their adhesion abilities. Cultures were first diluted to an O.D._600_ of 0.05 and 5 μL were used to inoculate 95 μL of fresh BHI broth supplemented with 0.1% sucrose to measure sucrose dependent adhesion or 0.1% glucose to measure sucrose-independent adhesion in a 96-well plate, in triplicate. The plates were then incubated without shaking at 37 °C for 24 and 48 h with 5% CO_2_. After 24 h, the media was removed, and the plate was washed with distilled water. 200 μL of a 1% crystal violet solution was then added to the washed plate and left to incubate for 2 h. Plates were then washed with water three times and left to dry overnight. The following day, 200 μL acetic acid was used to release the crystal violet from adhered cells and then was read at 595 nm. Each population was plated in three separate wells to acquire three independent measurements for each population.

### Genome sequencing

Chromosomal DNA was extracted from the pellets originating from the 21-, 42–63- and 100-day samples for all eight evolved populations and the ancestral, using the E.Z.N.A. bacterial DNA extraction kit from Omega Biotek® as per the manufacturer’s protocol. Eluted DNA was then quantified using the QuantiFluor® dsDNA system (Promega). 300 ng of purified genomic DNA was used to prepare DNA libraries using the Nextera XT DNA library prep kit (Illumina). Samples were then sequenced on an Illumina miSEQ sequencing platform with depth of coverage ranging from ~100X to ~200X, with most exceeding 150X coverage. Sequence alignment and variant calling from the samples was achieved by use of the *breseq* 0.30.0 pipeline^75^. The breseq pipeline uses three types of evidence to predict mutations, read alignments (RA), missing coverage (MC), and new junctions (JC), and any reads that indicate a difference between the sample and the reference genome that cannot be resolved to describe precise genetic changes are listed as “unassigned.” These unassigned reads are not described nor interpreted here.

### Statistics

All phenotypic data was plotted in GraphPad Prism ® Version 9.2.0. Statistics for pairwise comparisons between ancestral populations and each individual treatment population were calculated using an unpaired T-test. Significance is defined by the two-tailed p-value using an *. * ≤ 0.05, ** ≤ 0.01, *** ≤ 0.001 and **** ≤ 0.0001. Linear regression was used to compare the number of accumulated mutations for each normal gravity population and each simulated microgravity population. General linear analysis in SPSS was used to compare the normal gravity and simulated microgravity populations. Finally, chi-squared analysis using SPSS was used to compare the genomic variants to determine the effect of environment on selection and a simple linear regression was performed in GraphPad Prism with a 95% confidence interval to determine the significance in the slope for reporting of accumulated mutations in both normal gravity and simulated microgravity.

### Reporting summary

Further information on research design is available in the Nature Research Reporting Summary linked to this article.

## Results

### DNA resequencing

Following the 100-day EE study, we assessed the genetic changes that arose in response to both normal gravity and simulated microgravity adaptation by whole genome DNA resequencing at multiple time points (21- (~294 generations), 42- (~588 generations), 63- (~882 generations) and 100-days (~1400 generations)). The *S. mutans* NCTC 10449 genome used in this study (Smutans_NCTC10449.gbk [https://www.ncbi.nlm.nih.gov/nuccore/NZ_LS483349.1]) is poorly annotated and characterized therefore, many gene functions are based on those with high sequence homology to *S. mutans* UA159 genes (SMU annotated genes)^76^. Alignment and variant calling were conducted using breseq 33.2^75^. Coverage distribution plots of mapped reads output from breseq showed a depth of coverage for unique positions ranging from ~150x to over 200x with most showing ~200x coverage. This far exceeds the minimum coverage for true variant calling of ~50x for reference guided assembly^77^. All sequencing data with frequencies of mutation (*f*) above 0.1 are summarized in Supplementary Table 1. The datasets generated and analyzed in this study can be found through the NCBI BioProject database under Bioproject number PRJNA759625, accession numbers SAMN23239391- SAMN23239423 [https://www.ncbi.nlm.nih.gov/sra/?term=PRJNA759625].

We began by sequencing the ancestral population (NCTC 10449 [https://www.ncbi.nlm.nih.gov/nuccore/NZ_LS483349.1]) and identified three variations when comparing it to the reference database sequence, this includes a SNP (I365S (ATT → AGT)) in DQM59_RS03765, a UDP-N-acetylglucosamine 2-epimerase, and an insertion in both DQM59_RS03875 a TIGR01906 family membrane protein and in the intergenic region between *trxB* ← / ← DQM59_RS08210, a thioredoxin-disulfide reductase and a DUF4059 family protein respectively. Therefore, these sweeps (*f* = 1.000*)* are not the result of our adaptation studies (Supplementary Table 1, highlighted in yellow).

### Shared-adaptation

Over the course of the 100-day experiment, variants arose that appeared in both the normal and simulated microgravity treatments (Supplementary Table 1 - orange). On day 21, there were five variants that appeared in the intergenic region between an AAA-domain containing protein and a dihydrolipoyl dehydrogenase. These were detected in at least one replicate in each treatment, all with similar frequencies. At day 42, there were 3 shared variants, two of them in the same intergenic region above, and a new shared variant which appeared in a C3-glycoprotein degrading protease (C3-GDP) which, had initially been selected for in the simulated microgravity populations at day 21 (Table 1). Notably, the single normal gravity variant appeared at very low frequency (0.089) while at high frequency in two simulated microgravity populations (0.580 and 0.882); indicating that this variant could have been playing a role in simulated microgravity adaptation. At day 63, there were again three shared variants, only one of the intergenic variants remained, the C3-GDP variant had swept (*f* = 1.000) in one replicate of simulated microgravity while remaining at low frequency (0.087) in normal gravity. Again, supporting an adaptive role for this variant in simulated microgravity. There was also a new shared variant in DQM59_RS07180, *ptsH* (phosphocarrier protein HPr), that was observed at low frequency in two normal gravity replicates and at high frequency (with one sweep *f* = 1.000) in the simulated microgravity (Table 1). Finally at day 100 there were six shared variants. One in the same intergenic region between an AAA-domain containing protein and a dihydrolipoyl dehydrogenase, one in *ptsH*, repeating the pattern from day 63, two in the C3-GDP with selective sweeps in both normal gravity and simulated microgravity (Table 1), and two new variants DQM_RS03695 (hypothetical protein) and DQM_RSO7235 (N-acetyl-gamma-glutamyl-phosphate reductase) at intermediate frequencies.

**Table 1:** Frequency of mutations detected earlier in simulated microgravity populations.

Gene	Day	NG1	NG2	NG3	NG4	MG1	MG2	MG3	MG4	Annotation
SMU_399	21					0.10				S112Y
C3-GDP							0.06	0.26		C 302–313/759
									0.14	C 24–36/759
						0.10				E58*
	42	0.29								C276–514/759
		0.09					0.58	0.88		C 302–313/759
						0.47				S112Y
						0.09				E58*
							0.10			E221*
									0.58	C 538/759
	63	0.30								C 276–514/759
		0.08					0.85	0.63	1.00	C 302–313/759
						1.00				S112Y
				0.11						Q130*
								0.09		C 664/759
								0.23		C 29/759
	100	1.00		1.00			1.00			C 7/759
			1.00		0.26		0.17	0.66	1.00	C 302–313/759
						0.86				S112Y
						0.10				E58*
							0.09			E221*
ptsH	42					0.19	0.50			G54A
	63		0.33		0.21	0.43	0.72	0.34	1.00	G54V
	100				0.06	0.06			0.93	G54V
rex	42					0.13				Q202*
						0.06				T155K/G65S/D52N/
										R51L/S46L^a^
							0.22			Y66C
								0.83		Q193P
									0.66	A33E
	63	0.25								R51H
			0.07							R14H
				0.26						T155R
				0.61						T48I
					0.05					G60S
					0.10					A47V
						0.08				G57S
						0.37				A33E
							0.13			Y66C
								0.53		Q193P
								0.05		A115E
	100				0.20					R51L
							0.58			Y55D

*Indicates premature stop codons.

C indicates in the coding region.

^a^MG1 acquired 5 different mutations all at an *f* ~0.06.

### Adaptation in normal gravity

Sequencing data also showed adaptive variants unique to the normal gravity populations (Supplementary Table 1 - blue). At day 21 there were 3 unique non-synonymous (NS) SNP variants ranging from *f* = 0.076–0.265 in all four normal gravity populations in two genes. At day 42, there were 22 unique polymorphisms (*f* = 0.051–0.744), of these, 16 were NS SNPs, 3 were synonymous (S) SNPs, and the rest were in the intergenic/coding regions. At day 63 there were 58 unique variants (*f* = 0.051–0.711), 35 were NS SNPs, 14 S SNPs, and 9 were intergenic/coding. Finally at day 100, there were 45 unique polymorphisms, of which 32 were NS SNPs, 2 were S SNPs, and 11 were intergenic/coding. Of these 45, three genes were shared among multiple populations and had variants solely present in normal gravity (Tables 2 and 3). This includes variants in, a DUF1003 domain-containing protein (NG1-E195G and R199C, NG2-R181C and S231R, NG3-Q100* and R140*), and the Cof-type HAD-IIB family hydrolase (NG3-E244* and NG4-G54A) and *sprV* (NG1-K15N and NG3-F52V).

**Table 2:** Frequency of mutations detected earlier in normal gravity populations.

Gene	Day	NG1	NG2	NG3	NG4	MG1	MG2	MG3	MG4	Annotation
pknB	42			0.05						G174C
				0.13						R45C
					0.11					Y475*
	63	0.34								Y561*
		0.11								D83H
				0.28	0.26					R45C
							0.08			D78E
									0.86	R258C
	100				0.13					F58S
					0.35					R45C
						0.16				I2S
									0.09	R258C
									0.60	G19A
DUF1003 domain containing protein	21	0.07	0.14							L168I
	42	0.07								R181C
		0.10								S231R
									1	A157V
	100	0.65								E195G
		0.17								R199C
			0.93							R181C
			0.94							S231R
				0.28						Q100*
				0.54						R140*
					0.24			0.52		D96E
						0.12				K102Q
						0.47				T235R
							0.11			L168I
								0.11		A166S
								0.13		A166D
									0.92	A157V
DQM59_RS01880	100			0.90						E244*
Cof‑type HAD‑IIB family hydrolase										
					0.26			0.53		A242E
					0.15					G54A
				0.90						E244*

*Indicates a STOP codon.

**Table 3:** Frequency of variants unique to normal gravity.

Day	NG1	NG2	NG3	NG4	Annotation	Gene
21			0.11	0.24	F52V	*DQM59_RS10195* ← *(sprV)*
			0.27	0.18	H21N	*DQM59_RS10195* ← *(sprV)*
42			0.38	0.14	Q31*	*DQM59_RS04330* → (*ridA)*
	0.08				R34S	*DQM59_RS10195* ← *(sprV)*
		0.05			E72K	*DQM59_RS10195* ← *(sprV)*
		0.24			K15N	*DQM59_RS10195* ← *(sprV)*
			0.08	0.17	F52V	*DQM59_RS10195* ← *(sprV)*
			0.12	0.13	H21N	*DQM59_RS10195* ← *(sprV)*
		0.10			T104A	*vicK*
	0.74	0.15			C 2051–2131/4689	*spaP* ←
				0.08	S721L	*spaP* ←
	0.35				K373N	*DQM59_RS07770* ←(*murD*)
63			0.28	0.46	Q31*	*DQM59_RS04330* → (*ridA*)
		0.71			F219L	*DQM59_RS06715* ←
	0.22				K373N	*DQM59_RS07770* ←(*murD*)
	0.25				R34S	*DQM59_RS10195 ← (sprV)*
	0.08				H21Y	*DQM59_RS10195* ← *(sprV)*
		0.54			K15N	*DQM59_RS10195* ← *(sprV)*
			0.58		F52L	*DQM59_RS10195* ← *(sprV)*
			0.10		F52V	*DQM59_RS10195* ← *(sprV)*
				0.36	H21N	*DQM59_RS10195* ← *(sprV)*
					E55D	*DQM59_RS10195* ← *(sprV)*
	0.22				K7Q	*DQM59_RS02455* →
	0.59	0.05			C 2051–2131/4689	*spaP* ←
			0.08		S721L	*spaP* ←
				0.09	N917N	*spaP* ←
		0.21		0.05	R205C	*DQM59_RS03385* →
100				0.55	Q31*	*DQM59_RS04330* → (*ridA*)
				0.33	I(+280/−96)	*DQM59_RS00680* → / → *DQM59_RS00685*
			0.53		F98V	*DQM59_RS03205* →
		1.00			I(−236/−20)	*acnA* ← / → *DQM59_RS07205*
		0.62			C 464–493/495	*DQM59_RS01355* →
		0.93			P169L	*DQM59_RS03185* →*(trkA)*
				0.18	S191*	*DQM59_RS07640* →*(lytS)*
				0.15	Q229*	*DQM59_RS07640* →*(lytS)*
	0.11				K15N	*DQM59_RS10195* ← *(sprV)*
			0.06		F52V	*DQM59_RS10195* ← *(sprV)*
			0.60		V81F	*vicK*
			0.38		I407F	*vicK*
				0.26	A237D	*vicK*
				0.20	S721L	*spaP* ←

*Indicates premature stop codons.

C indicates in the coding region.

I indicates intergenic region.

In addition, many unique variants were detected in a single normal gravity population (Table 3). The only one that was acquired early and maintained from one sequencing time point to the next was the Q31* mutation in a RidA family protein in NG4. This variant first arose in two populations (NG3; *f* = 0.381 and NG4; *f* = 0.138) by day 42 and was only maintained in NG4 (*f* = 0.547) at 100-days indicating these populations were likely outcompeted by clones encoding the wild-type gene sequence. NG2 acquired 3 high frequency mutations, this includes one sweep in the intergenic region between *acnA* and *nrdH* (*f* = 1.000), a 30 bp deletion in the coding region of the PTS sugar transporter, subunit IIB (*f* = 0.619) and a P169L mutation in *trkA* (*f* = 0.925). NG3 acquired two mutations at intermediate frequency, a F98V mutation in a CBS domain-containing protein (*f* = 0.527) and a P239S mutation in a HAD family hydrolase (*f* = 0.331). Finally, NG4 acquired ten mutations in nine different genes, three of the variants showed intermediate frequency including those detected in the RidA family protein, an intergenic mutation between an NAD(P)H-dependent oxidoreductase and a thiamine pyrophosphate-dependent dehydrogenase (*f* = 0.333) and two separate variants in *lytS* (S191* *f* = 0.181 and Q229* *f* = 0.150).

### Adaptation in simulated microgravity

Sequencing results showed that there were several variants unique to the simulated microgravity populations (Supplementary Table 1 - green). At day 21 there were 8 unique variants ranging from *f* = 0.063–0.333 in the four simulated microgravity populations, of these, 3 were NS SNPs and 5 were intergenic/coding. At day 42 there were 17 unique polymorphisms (*f* = 0.053–0.575), 14 were NS SNPs, 1 a S SNP, and two in the intergenic/coding regions. At day 63 there were 14 unique variants (*f* = 0.052–1.000), 11 were NS SNPs and 3 were intergenic/coding. Finally at day 100, there were 30 unique polymorphisms (*f* = 0.059–0.929), 24 were NS SNPs and 6 were intergenic/coding.

At 21 days, the most significant variant was an insertion in pseudogene DQM59_RS099330 that occurred in MG1. In addition, five different variants were detected in the C3-GDP for all four simulated microgravity populations (normal gravity populations did not acquire mutations in this gene until day 42, Table 1). By day 42, each of the simulated microgravity populations had a least one variant that had arisen to a frequency > 0.470. These occurred in four different genes: the redox-sensing transcriptional regulator *rex*, the phosphocarrier protein *ptsH*, the C3-GDP and an intergenic mutation between an AAA domain-containing protein and dihydrolipoyl dehydrogenase (Table 1 and Supplementary Table 1). At day 63, four unique mutations were detected at a frequency > 0.520 in the four replicates. These include: DQM59_RS01220 (DUF1033 family protein), *rex*, *pknB* (Stk1 family PASTA domain-containing Ser/Thr kinase) and the C3-GDP (Tables 1 and 2). Finally at day 100, 12 unique variants were detected in the four replicate populations (ten at frequencies > 0.400). Nine of these variants were NS SNPs and 3 were intergenic or within a pseudogene. At 100-days there were three genes that acquired mutations in multiple populations and carried variants which were unique to the simulated microgravity environment; this includes the DUF1003 domain-containing protein (MG1-K102Q and T235R, MG3-A166S and A166D and MG4-A157V), *pknB* (MG1-IS2, MG4-R258C and G19A) and C3-GDP (MG1-S112Y and E58*, MG2-E221* and MG4–14bp and a 6 bp coding region insertion) (Tables 1 and 2). These are in addition to the single variants in each population at 100-days (Table 4). Here, MG1 adapted two high frequency mutations, one an intergenic mutation in polymerase subunit delta (*f* = 0.836) and a G88A mutation (*f* = 0.877) in *rpoC* in addition to three low frequency mutations, one in the membrane protein DQM59_RS00535 (G77R *f* = 0.199), one in the ABC transporter permease *vex3* (M267L *f* = 0.130) and one in the glutathione-disulfide reductase *gorA* (E251* *f* = 0.114). MG2 had a low frequency (*f* = 0.154) W443* mutation in *vicK*. MG3 carried four low frequency unique variants with *f* = 0.150 in *trkB* (V96G), two in *lepA* (D80A and L81F) and one 114 bp deletion in the coding region of *spaP*. In addition, MG3 had three intermediate frequency variants, *f* = 0.315 in a pseudogene, a Y88C mutation (*f* = 0.370) in *sprV* and a Y55D mutation (*f* = 0.582) in *rex*. MG4 had only one unique low frequency mutation (*f* = 0.141) in the intergenic region of *asnS* and a hypothetical protein.

**Table 4:** Frequency of variants unique to simulated microgravity.

Day	MG1	MG2	MG3	MG4	Annotation	Description
42	0.06				G88A	*rpoC*
63		0.20			G123C	*ccpA* →
	0.12				Y636*	*DQM59_RS06275* ←
		0.12			A78V	*DQM59_RS04610* →*(eno)*
	1.00				G88A	*rpoC*
		0.06			E105G	*rpoC*
		0.12			E55D	*DQM59_RS10195* ← *(sprV)*
			0.11		L22I	*ylqF* ←
100				0.14	intergenic (+181/+120)	*asnS* → / ← *DQM59_RS04320*
		0.20			G77R	*DQM59_RS00400* →
	0.84				intergenic (−381/−244)	*DQM59_RS00535* ← / → *DQM59_RS00540*
			0.15		V96G	*DQM59_RS03190* →*(trkB)*
			0.15		D80A	*DQM59_RS03980* →(*lepA)*
			0.15		L81F (	*DQM59_RS03980* →(*lepA)*
		0.13			M267L	*DQM59_RS05315* ←*(vex3)*
			0.32		pseudogene (217/304 nt)	*DQM59_RS08775* ←
	0.88				G88A	*rpoC*
		0.65			H21N	*DQM59_RS10195* ← *(sprV)*
			0.37		Y88C	*DQM59_RS10195* ← *(sprV)*
			0.41		A237D	*vicK*
		0.15			W443*	*vicK*
			0.19		coding (1588–1701/4689)	*spaP* ←
		0.11			E251*	*gorA* ←

*Indicates premature stop codons.

C indicates in the coding region.

I indicates intergenic region.

### All populations show changes in adhesion

**Fig. 2:**
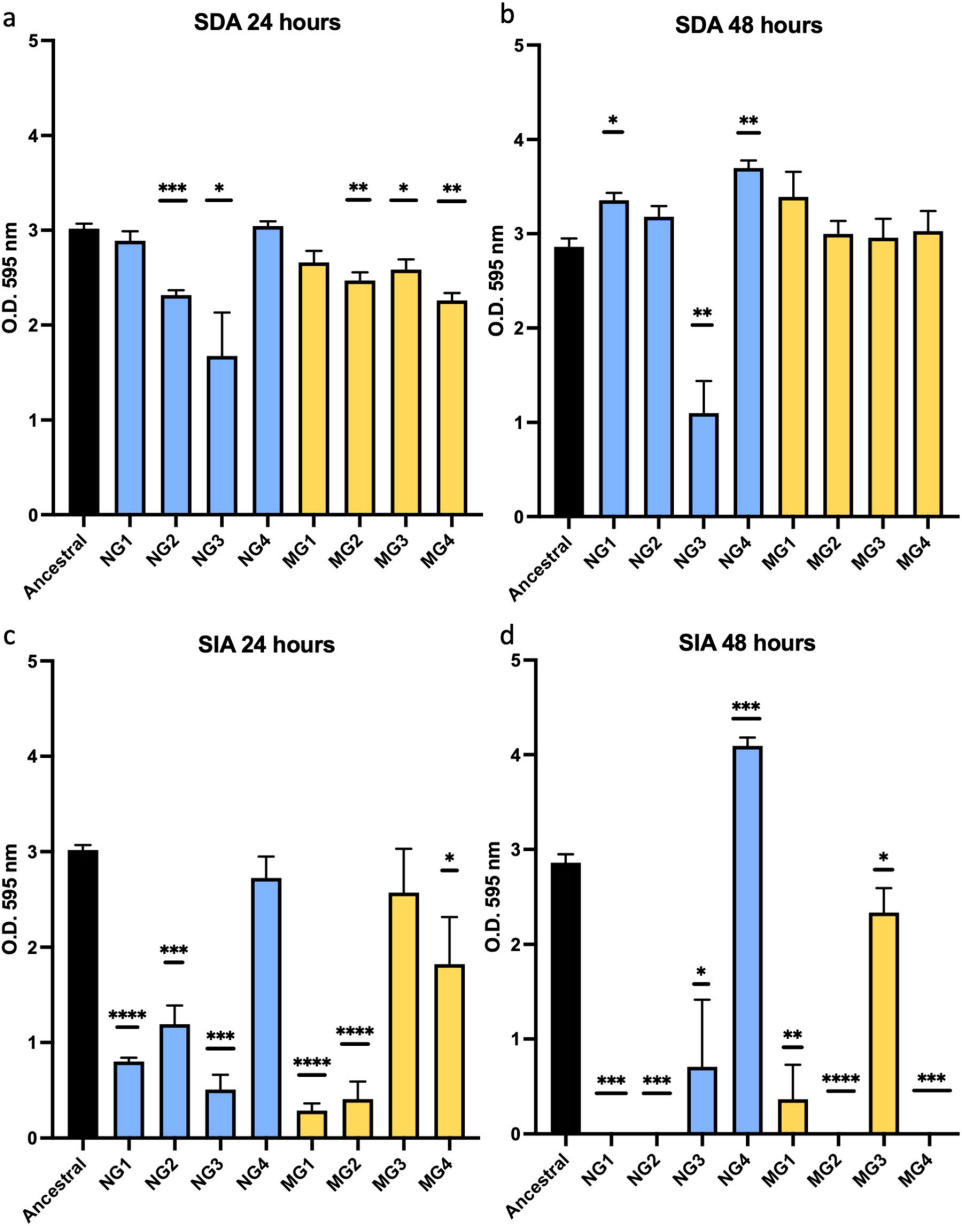
Adhesion phenotypes vary between biological replicates. Sucrose-dependent (SDA) was assessed after (**a**) 24- and (**b**) 48 h of static growth in BHI supplemented with 0.1% sucrose using the 100-day populations. Sucrose-independent adhesion (SIA) was assessed after (**c**) 24- and (**d**) 48 h of static growth in BHI supplemented with 0.1% glucose using the 100-day populations. Data was plotted in GraphPad Prism ® 9.2.0 and unpaired *t*-tests with 95% confidence were used to calculate significant differences for pairwise comparisons between the ancestral and each treatment populations. Error bars are s.e.m. and significance is reported as a two-tailed p-value where *p ≤ 0.05, **p ≤ 0.01, ***p ≤ 0.001 and ****p ≤ 0.0001.

*Streptococcus* pathogenicity is reliant on the ability to adhere to the tooth surface and form biofilms commonly known as plaque. They do so by forming aggregates that fuse with the tooth pellicle via two independent mechanisms: sucrose-dependent and sucrose-independent adhesion. The former is adequate for initial adherence, but the latter is required for virulence and pathogenicity^52^. We therefore analyzed both sucrose dependent (SDA) and sucrose-independent adhesion (SIA) for the four 100-day adapted normal gravity populations (NG1–4) and the four 100-day simulated microgravity adapted populations (MG1–4) and performed pairwise comparisons to the phenotypes exhibited by the ancestral population (Fig. 2). SDA was assessed by inoculating 96-well plates containing BHI media supplemented with 0.1% sucrose with cultures from the HARVs diluted to a starting O.D._600_ of 0.05 and left to incubate without shaking for both 24 and 48 h. Comparisons with the ancestral population show a decrease in SDA in two of the normal gravity populations (NG2 and NG3) and a small decrease in three of the simulated microgravity populations (MG2–4) when grown for 24 h (Fig. 2a). By 48 h, all populations had restored similar adhesion levels (NG2, MG1–4) or higher (NG1 and NG4) than to that of the ancestral, except for NG3 which continued to show a significant deficit in SDA even after 48 h (Fig. 2b).

The differences in SIA were more dramatic (Fig. 2c, d). Here, 96-well plates were filled with BHI supplement with 0.1% glucose. BHI broth does contain a low concentration of glucose (2 g L^−1^) and therefore the +0.1% is in addition to that already present in the medium. After 24 h in +0.1% glucose we observed a significant decrease in SIA for NG1–3, MG1, MG2 and MG4. After 48 h NG1, NG2, MG2 and MG4 had complete loss of SIA, whereas NG3 and MG1 had similar levels to what was observed after 24 h. MG3 showed a small decrease at 48 h from that of the ancestral and NG4 showed a significant increase in SIA after 48 h. Apart from NG4 and MG3, SIA is almost completely lost in both environments. NG4 shows the lowest amount of variation, with no significant change for both SIA and SDA at 24 h and a slight increase in both phenotypes after 48 h. Similarly, MG3 only exhibits a small decrease in SDA at 24 h and SIA at 48 h when compared to the ancestral. We also evaluated both SIA and SDA at the 21-day timepoint and for SIA, all four normal gravity and all four simulated microgravity populations showed no significant variation from that of the ancestral after both 24- and 48 h (Supplementary Fig. 1A, B). For SDA, NG2 and NG3 showed a slight increase over the ancestral but NG1, NG4 and MG1–4 all show no variation from the ancestral after both 24- and 48 h (Supplementary Fig. 1C, D). This data is important to show that the 100-day phenotypic data is specific for this time point and not for any random amount of time in the HARV. Overall, it is difficult to predict the influence of the selection environment on this phenotype as changes are observed in both conditions, but it is important to note that phenotypes do vary greatly between biological replicates.

### Acid tolerance is specific to the adaptive environment

**Fig. 3:**
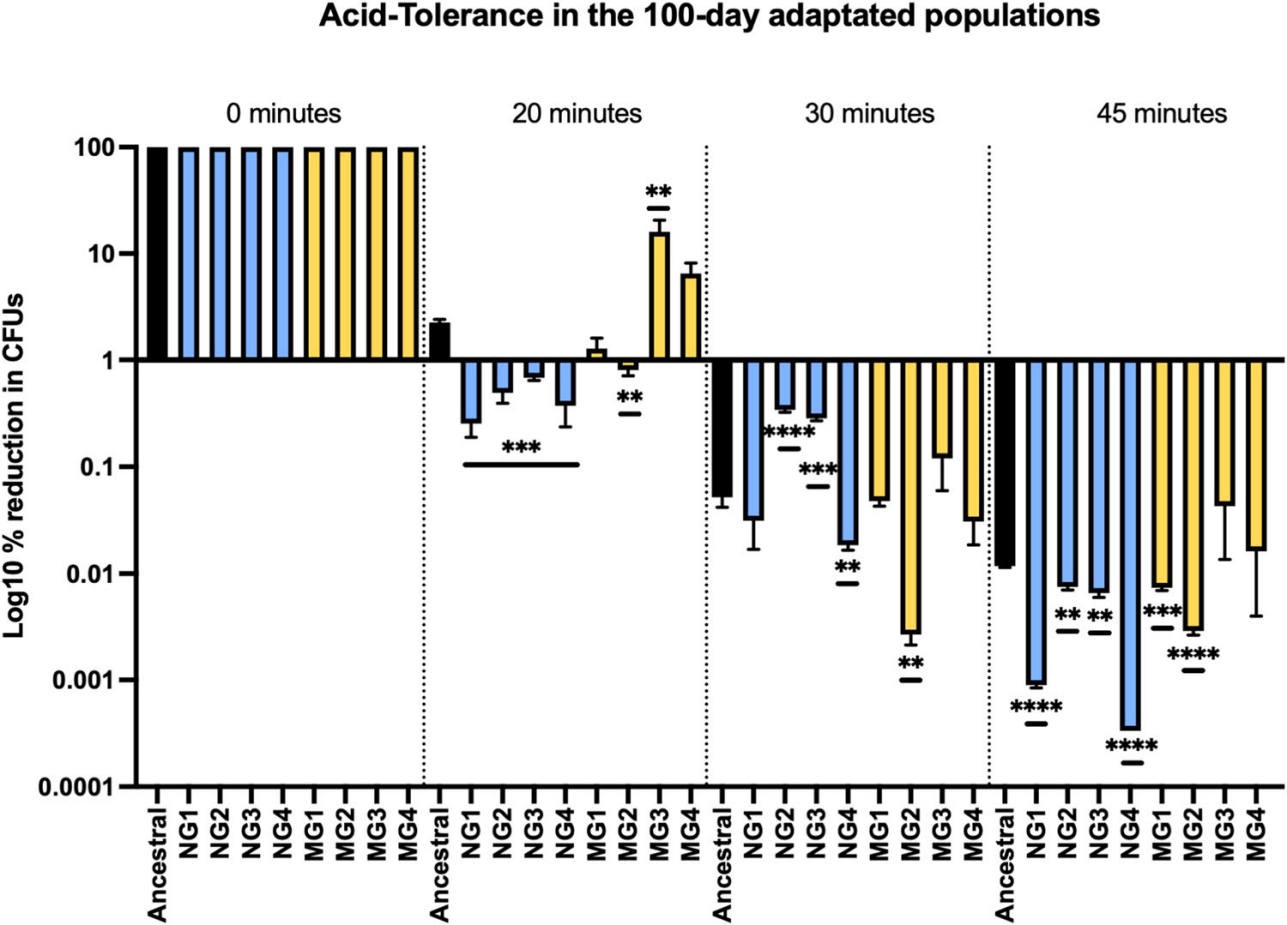
Acid-tolerance changes as a result of simulated microgravity. We assessed changes in acid-tolerance after adaptation to both normal (blue) and simulated microgravity (yellow) and compared it to that of the ancestral (black). The 100-day populations were exposed to an acidic environment (glycine pH 2.8) for 0, 10, 20, and 45 min, rescued, serial diluted and CFUs were counted after 48 h of growth on BHI agar plates. The CFU counts at the 0-time point were then normalized to 100% and the % reduction was then calculated by dividing the CFU count at the indicated time points by the CFU count at 0. The values were then plotted on a log10 scale to visualize the data. Data was plotted in GraphPad Prism ® 9.2.0 and unpaired t-tests with 95% confidence were used to calculate significant differences for pairwise comparisons between the ancestral and each treatment populations. Error bars are s.e.m. and significance is reported as a two-tailed p-value where *p ≤ 0.05, **p ≤ 0.01, ***p ≤ 0.001 and ****p ≤ 0.0001.

Sustained plaque below a pH of 5.4 favors demineralization of tooth enamel and development of dental caries, as a result, *S. mutans* uses proton pumps to maintain intracellular pH levels and tolerate growth in acidic environments while part of human dental plaque^78^. We therefore assessed changes in acid tolerance by exposing the ancestral populations, along with each of the eight evolved populations to acidic environments for 0, 20, 30 and 45 min and counted CFUs to evaluate survival and therefore their ability to tolerate low pHs (Fig. 3). It is important to reiterate that our cells are planktonically grown and not in biofilms as they would be in plaque. General linear analysis shows a highly significant difference (*p* < 0.001) between the normal gravity and simulated microgravity populations when the 0-time point is omitted indicating that adaptation of this phenotype is specific to the environment. Pairwise comparisons show that generally, acid tolerance decreases as a result of adaptation to normal gravity when compared to the ancestral population. Most notably, all four normal gravity populations show a significant reduction in tolerance to low pH after exposure for both 20 and 45 min. Simulated microgravity replicates on the other hand, tend to be more variable in their adaptive response and overall, quite similar to the ancestral. MG1 shows no significant difference at both 20 and 30 min, with a slight reduction after 45 min when compared to the ancestral. MG2 shows a reduction in acid tolerance at all time points whereas MG3 displays an increase in acid tolerance at 20 min, then shows no change as compared to the ancestral after 30 and 45 min. MG4 shows no change in acid tolerance at all time points. However, this phenotype is usually required while *S. mutans* resides as part of the biofilm community (plaque), therefore a general decrease in acid tolerance is not surprising for these planktonic populations. We also collected acid tolerance data for the 21-, 42-, 63- populations (Supplementary Fig. 2a–d). The early data varies at each timepoint, with the earliest timepoints showing the greatest differences from the ancestral populations. Again, reiterating that the 100-day data is specific for this time point.

### Antibiotic susceptibility

Many others have shown changes in antibiotic susceptibility as a result of exposure to simulated microgravity^25^. Here we exposed *S. mutans* to six antibiotics traditionally used to treat dental infections, this included: amoxicillin, penicillin, erythromycin, vancomycin, methicillin, and clindamycin using Etest® strips and found that after 100-days of adaptation, normal gravity populations showed no change in antibiotic susceptibility when compared to the ancestral to any of the six antibiotics tested (Supplementary Fig. 4). Generally, the simulated microgravity populations showed no change to the tested antibiotics with the exception of a small increase in erythromycin susceptibility for MG4 and an increase in resistance in MG1 towards clindamycin.

## Discussion

The initial goal of this study was to determine the genomic foundations of adaptation in *Streptococcus mutans* to simulated microgravity. Our data shows that after 100-days (~1400 generations) of adaptation, planktonic *S. mutans* do carry specific variants that can be uniquely attributed to simulated microgravity populations. Our data also stresses the importance of biological replicates, as none of the unique variants were present in all four simulated microgravity populations. In addition, we show the necessity of the normal gravity controls and reporting the frequency of mutation as many of the unique variants were detected in common genes between normal gravity and simulated microgravity and often at very different frequencies, stressing the importance of these data in assigning and validating the roles these genes may be playing in the adaptive response to simulated microgravity.

**Fig. 4:**
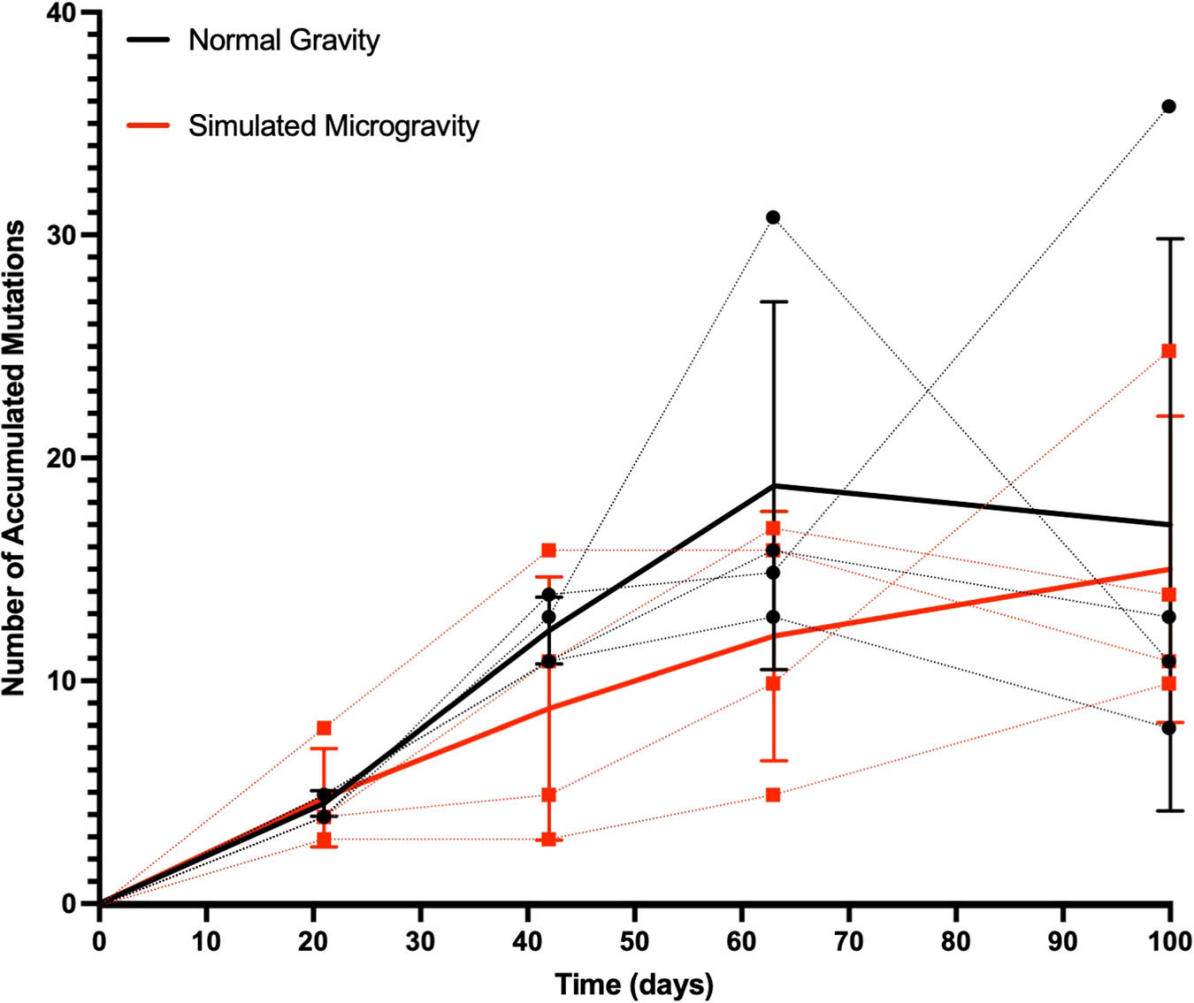
Populations in simulated microgravity accumulate less mutations. The number of accumulated mutations were plotted for each normal gravity population (blue dotted lines) and each simulated microgravity population (yellow dotted lines) for each sequencing time point. The mean (solid lines) and standard deviation (error bars) were then plotted for each environment. Linear regression determined slopes were significantly different (*p* < 0.0001). Data was plotted and statistical analysis were performed in GraphPad Prism ® 9.2.0. significance is reported as a two-tailed p-value where *p ≤ 0.05, **p ≤ 0.01, ***p ≤ 0.001 and ****p ≤ 0.0001.

When comparing the genomic variants observed in normal gravity to those in simulated microgravity after 100 days of selection, as stated, 13 variants were shared by both normal gravity and simulated microgravity; 43 were unique to normal gravity, and 29 were unique to simulated microgravity, for a total of 86 variants (chi-squared test showed that χ^2^ = 421, *p* < 0.0001). If there had been no effect of environment on selection, we would expect the numbers of variants shared by both, normal gravity alone, and simulated microgravity alone to be equal (~29 each). Figure 4 demonstrates the number of accumulated mutations in each environment. These data show that after 100-days in either normal gravity or simulated microgravity, both genomes are still in flux. Also, in simulated microgravity, *S. mutans* accumulates mutations at a rate significantly slower (linear regression of the slopes show *p* < 0.0001) than normal gravity. There is also significant variance between the populations at the different time points indicated by the standard deviation of each environment at each timepoint. We therefore suggest that simulated microgravity displays greater selection than normal gravity. There is no actual scale for measuring the strength of the selective environment and it is all relative to the fitness associated with the selective environment and can only be considered weak in making a comparison to a second environment. Therefore, the differences in these slopes showed that under our conditions, simulated microgravity is accumulating mutations at a lower rate than normal gravity. Mutation accumulation experiments generally show that more mutations can be observed in “weaker” selecting environments, this is due to the fact that most mutations are deleterious^79–82^. Thus, we can conclude that normal gravity was “weaker” than simulated microgravity.

Over 100-days of selection in both environments we also see refinement of many of the mutations that are present in the genomes of our sequenced populations. For example, in the intergenic region preceding the dihydrolipoyl dehydrogenase (*lpdA*) at day 21- there were five different variants present at different frequencies across all four normal gravity populations and all four simulated microgravity populations. This was reduced to only three variants across all four normal gravity populations and three simulated microgravity populations by day 42 with an increase in frequency across the board. We see similar trends for 63- and 100-days and we would hypothesize that these persistent variants (+177/−52 and +49/−180), are outcompeting the minor variants (+159/−170, +178/−51, +179/−50 and +184/−45). As expected, we observed this exact trend for all persistent variants identified in our sequencing data.

**Fig. 5:**
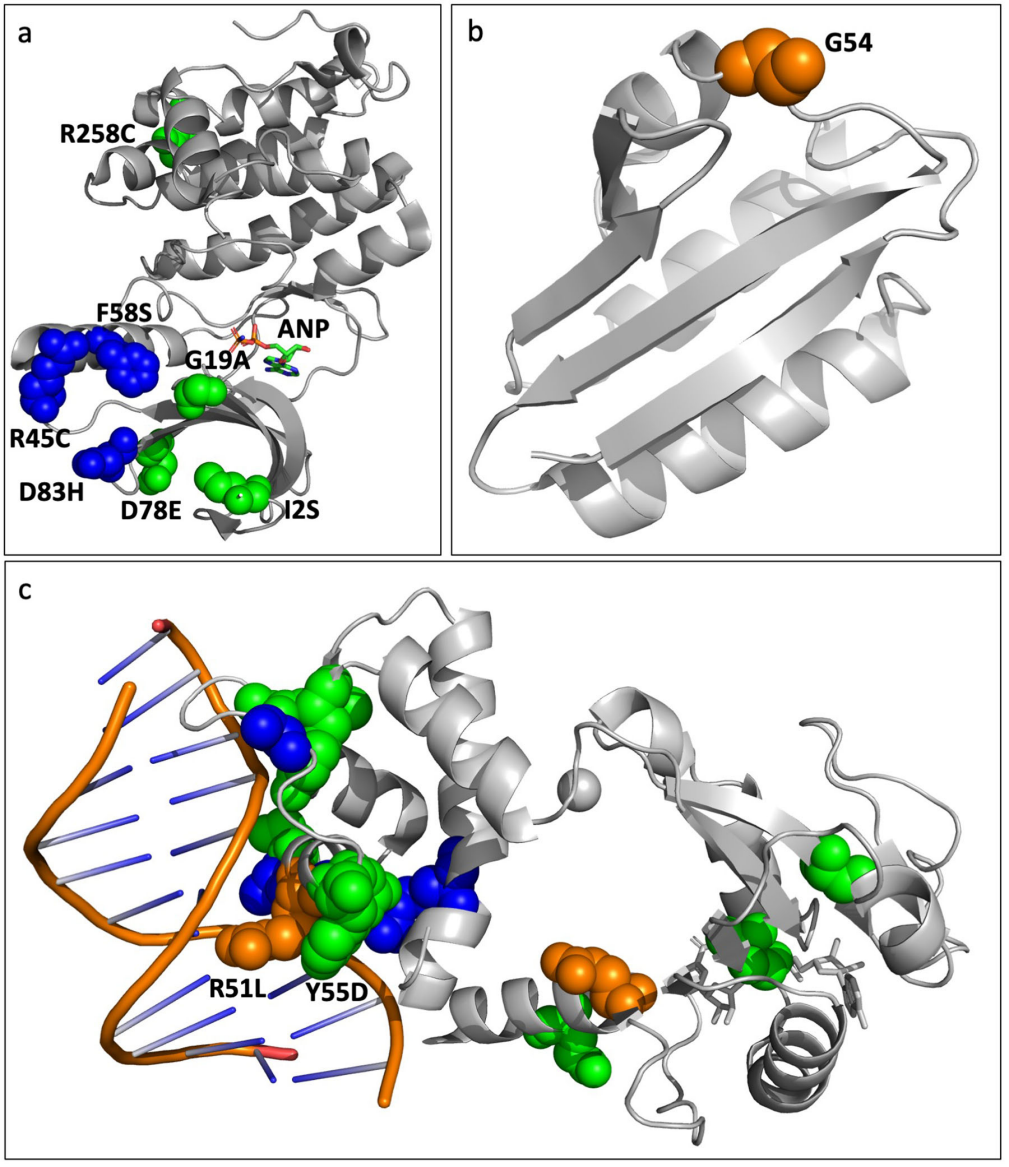
Unique genomic variants map to distinct regions of adapted genes. **a** *pknB* acquired mutations that were unique to either simulated microgravity (green) or normal gravity (blue). Here we mapped all mutations detected (excluding nonsense mutations, as they lead to a premature stop codon which results in a shortened, and most often, nonfunctional protein product) at all four sequencing time points onto a homolog from *Staphylococcus aureus* (PDB 4EQM [https://www.rcsb.org/structure/4EQM])^83^ as there were no solved *Streptococcus* homologues in the database. **b** *ptsH* acquired the same SNP in both normal gravity and simulated microgravity (G54A/V-orange). This specific residue has been shown to be important for protein-protein interactions (PDB 1PTF [https://www.rcsb.org/structure/1PTF])^89^. **c** *rex* acquired a total of 12 unique variants in simulated microgravity (green), 5 unique variants in normal gravity (blue) with 3 variants in common (orange). Variants mapped onto the homologous *Streptococcus agalactiae* structure (PDB 3KET [https://www.rcsb.org/structure/3KET])^99^ show that they both interact directly with the DNA substrate. All structural figures were generated using The PyMOL™ Molecular Graphics System, Version 2.4.1, Schrödinger, LLC.

At 100-days, simulated microgravity treatments held unique variants in multiple populations in the DUF1003 domain containing protein SMU_1307c (MG1, 3 and 4), *pknB* (MG1, 4) and in the C3-GDP (MG1, 2 and 4). Normal gravity populations also held unique variants in both SMU_1307c (NG1, 2 and 3) and in *pknB* (NG4). It is therefore important to determine if these unique variants could be leading to different structural and functional outcomes in the resultant proteins that may be unique to the selection environment. SMU_1307c is an uncharacterized protein predicted to have two transmembrane (TM) segments (residues 112–136 and 148–171) (uniprot Q8DTM6 [https://www.uniprot.org/uniprot/Q8DTM6]). Most of the unique simulated microgravity mutations reside within these TM regions (3/5) whereas all the unique normal gravity variants (excluding nonsense mutations) are outside of these segments (4/4). Of the two variants in common between normal gravity and simulated microgravity, one lies within and the other outside of the TM segments. Overall indicating that there may be different structural outcomes between the normal gravity and simulated microgravity variants. PknB is a serine/threonine kinase also located in the bacterial membrane. In *Staphylococcus aureus* PknB has been shown to be important for full expression of pathogenesis and survival as it helps regulate purine biosynthesis, autolysis, its response to the human immune systems and antibiotic resistance^83^. We therefore mapped both the normal gravity and simulated microgravity variants on the homologous *S. aureus* structure (PDB 4EQM [https://www.rcsb.org/structure/4EQM]) as there were no *Streptococcus* homologs in the database. Of the eight different missense NS variants, six of the residues are conserved in *S. aureus* none of which are found in both normal gravity and simulated microgravity. Of the four missense variants unique to normal gravity, none mapped in regions that have been shown to be important for function (Fig. 5a – blue)^83^. The two nonsense mutations suggest that it may be beneficial in our normal gravity environment to disable the function of this protein. Of the four unique simulated microgravity variants (Fig. 5a – green), two (R258C and G19A) are highly conserved residues, with G19 being in a glycine-rich loop which is responsible for positioning the γ-phosphate of ATP required for signal transduction^83^. By 100-days only the G19A variant remains at high frequency (*f* = 0.600) in simulated microgravity populations along with two low frequency variants. In *Mycobacterium marinum pknB* is regulated by *sigH* and under simulated microgravity, *M. marimum* was shown to up-regulate *sigH*. Despite this, there was no detection of differential expression of *pknB*^84^. It is therefore possible that differential expression is not required as the gene itself acquires novel mutations to make it more efficient under simulated microgravity.

As previously stated, we also detected mutations in three genes earlier than they were detected in the normal gravity populations, indicating that these genes may play an important role in early adaptation to simulated microgravity. It is important to note that this information would have been lost had sequencing been performed at a single time point. Specifically, all four simulated microgravity populations had polymorphisms in SMU_399 a C3-GDP which is believed to help in immune evasion by impairing C3b deposition and surface binding of IgGs. It has also been shown to interact with competence genes that have been shown to play a role in adhesion while being negatively regulated by VicK (which also carried mutations in both normal gravity and simulated microgravity populations)^85^. By 100-days, mutations were maintained in three of the four simulated microgravity populations, two of which went to fixation. The specific variants were present in both normal gravity and simulated microgravity, but it is important to note the time it took to acquire these specific mutations in each of the selection environments. All four simulated microgravity populations carried C3-GDP mutations by 21-days and all four maintained variants into day 100, all at high frequency. One normal gravity population acquired mutations in this gene by 42-days and not until 100-days did all four normal gravity populations select variants. It is also worth noting that NG4 is the only variant to not acquire a high frequency mutation, and it is the only population to show a unique phenotype in both SIA and SDA when compared to the other populations. Others have identified changes in the *Streptococcal* C3-GDP in response to antibiotic resistance^86^ and fluoride resistance^87^ although, as this is the first adaptive experiment in a *Streptococcal* species to simulated microgravity, it is the first time that it has been associated with this type of environment.

Two simulated microgravity populations also held *ptsH* (SMU_674) mutations by day 42, these became high frequency variants by 63 days when intermediate frequency variants were first detected in two normal gravity populations. By 100-days there was a single low frequency (0.063) variant remaining in the normal gravity populations, while a high frequency variant (0.933) was maintained in MG4. The *ptsH* protein product, HPr, is a phosphocarrier protein and an essential component of the phosphoenolpyruvate phosphotransferase system (PTS) required for sugar metabolism and virulence^88^. G54 is highly conserved in most Hpr proteins^89–91^, therefore we mapped the G54A/G54V mutations onto the homologous HPr structure from *Streptococcus faecalis* (PDB 1PTF [https://www.rcsb.org/structure/1PTF], Fig. 5b). G54 is located at the center of an external surface loop suggested to play a role in protein-protein interactions. Again, the variant itself is not unique to simulated microgravity, just the time frame at which high frequency variants were first detected. Other simulated and true space flight experiments have also shown changes in expression of bacterial PTS systems. *Staphylococcus warneri* was shown to upregulate its PTS system during long-term space flight, which was suggested to enhance its resistance and adaptability to its environment^92^. In addition, changes in PTS regulation have been shown in *Serratia marcescens* (spaceflight)^93^*, Salmonella enteriditis* (spaceflight)^94^, *Yersinia pestis* (simulated microgravity)^95^ and *Salmonella enterica* serovar *typhimurium* which differentially expressed *ptsH* specifically (simulated microgravity)^96^. Most notably, *S. mutans* was shown to downregulate PTS genes in normal gravity when compared to simulated microgravity after 48 h of exposure by Orsini et al.^53^

Finally, mutations were detected early in all four simulated microgravity populations (42-days) in *rex*, a redox sensing transcriptional regulator that plays an important role in carbon metabolism, the oxidative stress response and biofilm formation^97,98^. Variants in all four normal gravity populations arose by day 63, although only two variants were maintained in MG2 and NG4 at 100-days. In total, 18 variants were detected across populations, all of which display a complete change in chemical composition of the resultant sidechain and when mapping those mutations on a homologous structure from *Streptococcus agalactiae*, most mutations (12/18) map to residues interacting directly with the DNA substrate in both normal gravity and simulated microgravity populations (PDB 3KET [https://www.rcsb.org/structure/3KET])^99^. Specifically, all unique normal gravity variants map to the DNA-binding region (Fig. 5c-blue) whereas both the shared (Fig. 5c-orange) and simulated microgravity unique (Fig. 5c-blue) variants are located both within and outside of the DNA-binding domain, as a result, it is more difficult to predict the effect that these will have on the structure and function of the protein. The two variants maintained at 100-days, R51L (NG4 *f* = 0.198) and Y55D (MG2 *f* = 0.582) both interact directly with the DNA, R51 with the backbone and Y55 with the nitrogenous base itself and the changes in chemical nature will likely reduce affinity for DNA in both variants. Orsini et al.^53^ detected differential expression in *rex* regulated genes in their 48 h simulated microgravity studies with *S. mutans* which, was consistent with what was also found in both *Staphylococcus aureus*^100^ and *Lactobacillus reuteri* (simulated microgravity)^101^. These studies suggest that this differential expression may be the result of the cell perceiving an alteration in its oxidation state. As our study shows mutations in the DNA binding domain of Rex, this is consistent with others finding differential expression of Rex regulated genes.

It is therefore worth suggesting that different conformations of these proteins have the potential to be beneficial in normal versus simulated microgravity^102^. In addition, there must be a difference in genes that become more flexible in their resultant protein structure in simulated microgravity while maintaining adequate or similar growth in normal gravity vs those mutations that enhance fitness in simulate microgravity and negatively affect fitness in normal gravity. Computational modeling of how these mutations impact protein conformation may provide a clue to whether this mechanism has any salience. In addition, directed mutational studies to test the specific mutations in normal versus simulated microgravity would be the definitive test of their adaptive significance.

Finally, we detected unique variants at 100-day: one in MG1 (SMU_96 the DNA‑directed RNA polymerase subunit delta), three in MG2 (the membrane protein, SMU_66, an ABC transporter permease and *gorA*), four in MG3 (*trkB*, two in *lepA* and in SMU_1232c a DUF1697 domain containing protein) and one in MG4 (intergenic region between an asparagine tRNA ligase and a hypothetical protein). Their importance remains to be elucidated and would require increasing the length of the experiment, this flux also continues to support that short-term experiments cannot truly predict the adaptive behavior of these organisms in outer space.

Along with genomic changes, we also observed changes in phenotypes associated with our adaptation experiment. SDA assays were the first time that these populations were exposed to sucrose, as a result SDA was most notably reduced in NG2 and NG3 as well as MG2–4 after 24 h. Ancestral phenotype was restored in most populations at 48 h except for in NG3 which continued to show a significant reduction in this phenotype. SIA on the other hand exhibited a dramatic decrease in adhesion abilities in NG1–3 and MG1, 2 and 4 after 24 h. By 48 h, SIA abilities were almost completely lost in two normal gravity and two simulated microgravity populations. Changes in these traits were not unique to simulated microgravity as they were also observed in the normal gravity controls. We do hypothesize that this phenotype in the normal gravity controls may be the result of subculturing practices. In normal gravity, the cells settle to the bottom of the HARV making it possible that they can adhere to the base of the vessel especially with its slow rotation^53^. During subculturing, we primarily removed the planktonic cells thereby selecting those that are not adhering and potentially leaving those with greater adherence abilities in the HARV. This combined with the fact that they were adapted in presence of glucose could account for this data. As cells cannot settle in the simulated microgravity HARVs we believe this to be a true adaptive phenotype. This will be elucidated as we perform future experiments using *S. mutans* biofilms. Orsini et al.^53^ also observed an increase in cellular aggregation, this may have been the result of a short-term acclimation response to the novel environment, we show here that after 100 days planktonic *S. mutans* loses its ability to effectively adhere to surfaces via SDA. As SDA is required for initial attachment, this may also influence these organisms’ ability to cause disease after long-term adaptation. After 24 h exposure to simulated microgravity Cheng et al.^54^ found that *S. mutans* showed an increase in acid tolerance abilities. In our study, adaptation to normal gravity showed a clear reduction in acid tolerance, while in simulated microgravity we found that these results are quite variable between biological replicates. *Salmonella* also consistently and reproducibly alters its acid tolerance response in simulated microgravity and this response is dependent on the presence of media containing phosphate ions^96^. BHI media also contains 2.5 g L^−1^ disodium phosphate and could potentially be influencing this change in acid tolerance under simulated microgravity in a similar fashion.

Our phenotypic data is further supported by Fig. 4 as the accumulation in mutations for our populations are still trending upwards indicating that they have yet to reach an “optimal genome”. This also indicates that they have not yet reached optimal phenotypes. Based on the results of the Long-term Evolution Experiment (LTEE), we also have reason to predict that in any environment, under which *S. mutans* (or any other bacterium) is undergoing adaptation that there will never be an “optimal survival/growth point” and that the population will continue to evolve improvements in fitness indefinitely^103^.

Finally, the 100-day normal gravity populations showed no change in antibiotic susceptibility when compared with the ancestral to any of the six traditional antibiotics tested in this study. Two simulated microgravity populations showed small changes towards two antibiotics (MG4 an increase in susceptibility to erythromycin and MG1 an increase in resistance to clindamycin) indicating that change is possible, but generally not observed. This data was consistent with what was found for a number of other bacterial species grown in simulated microgravity including: *Yersinia pestis*^104^, *Escherichia coli*^62^, *Staphylococcus aureus*^105^, *Vibrio natriegens*^106^ and *Klebsiella pneumoniae*^107^. As our data stands, we see no clear increase in assessed virulence phenotypes (acid tolerance, adhesion nor antibiotic susceptibility) when planktonic *S. mutans* is adapted to simulated microgravity. Moving forward, as biofilm formation is required for pathogenesis, it will now be important to evaluate the adaptive response of *S. mutans* biofilm as a result of long-term exposure to simulated microgravity.

Overall, our data makes it clear that it is imperative to perform these adaptation experiments using multiple replicates over more than a few generations. Furthermore, our data is confounded by sequencing at multiple time points as mutation accumulation in both the normal gravity and simulated microgravity populations is still in an upward flux, making it hard to conclude the importance of many mutations in adaptation specifically for simulated microgravity. We show that each of our four simulated microgravity populations do have mutations in common, but overall, appear to be on their own independent evolutionary trajectory, each with its own unique set of mutations in genes that span a variety of biological processes as they continue to improve fitness. This can only be captured by having multiple biological replicates and to not overstate the impact of observed mutations detected in the sequencing data. It is also important to note that adaptation is occurring in response to all the culture conditions and the evolution of side-by-side normal gravity populations is essential in tying mutations directly to simulated microgravity. As our data concludes that for *S. mutans*, simulated microgravity is overall a weak selection environment, albeit greater than normal gravity evidenced by its reduction in accumulated mutation rate (yet significantly higher than a strong selection environment), population replicates become even more important in making connections between mutations and their role in adaptation. Some of the identified mutations evolved in both normal gravity and simulated microgravity environments indicating that they may be the result of adaptation to the culture conditions excluding the selective pressure of simulated microgravity. Thereby again stressing the importance of including normal gravity controls in these types of experiments. Finally, since we conducted sequencing experiments at multiple time points, we were able to observe the transitional variants in both normal gravity and simulated microgravity populations, in addition, we verified their frequency of mutation prior to assigning relevance to mutations and concluding the role a gene may play during adaptation. The sequencing results here show that the acquisition and loss of mutations in the genome is still in flux after 100-days of adaptation, had we conducted sequencing at a single time point, it would have been easy to make assumptions of mechanisms used to adapt to simulated microgravity. This also suggests that in space, microgravity may not be the main driver of evolution and moving forward, it will be important to factor in the other environmental conditions encountered by humans in space. By comparing simulated microgravity data to both normal gravity and ancestral data, having four biological replicates in each environment, sequencing at multiple time points and reporting the frequency of mutation; we are setting a new minimum standard for the field as the literature continues to grow and to reinforce accurate and reliable interpretations of the data obtained from such experiments. Finally, it is important to note that using rotating wall vessels to simulate microgravity does not actually change the magnitude of Earth’s gravity, rather they generate “functional near weightlessness” from the perspective of the organism growing in the vessel. Using these simulation devices have provided great insight into gravity-dependent changes however, they do produce artifacts that may distort desired microgravity effects^64^. Therefore, moving forward, it will be essential to perform comparative studies in real low gravity environments.

## Supplementary information

Supplementary Information

Reporting Summary

## Data Availability

The datasets generated and analyzed in this study can be found through the NCBI BioProject database (http://www.ncbi.nlm.gov/bioproject/) under Bioproject number PRJNA759625, accession numbers SAMN23239391- SAMN23239423 [https://www.ncbi.nlm.nih.gov/sra/?term=PRJNA759625].
