# Supplementary material for: Adaptation to simulated microgravity in Streptococcus mutans
Source: NPJ Microgravity. 2022 Jun 2;8:17. doi: 10.1038/s41526-022-00205-8 (PMC9163064; doi:10.1038/s41526-022-00205-8)
Supplement: Supplementary file 1 — Supplementary Information [file 41526_2022_205_MOESM1_ESM.pdf]

Supplementary Information

Adaptation to simulated microgravity in *Streptococcus mutans*

Mizpha C. Fernander, Paris K. Parsons, Billal Khaled, Amina Bradley, Joseph L. Graves Jr., and Misty D. Thomas

Supplementary Table 1 - Summary of mutations. (p. 2-7)

Supplementary Figure 1 – Adhesion after 21-days shows little change. (p. 8)

Supplementary Figure 2 – The acid tolerance fluctuates through adaptation**.** (p. 9)

Supplementary Figure 3- 48-hour growth curve. (p. 10)

Supplementary Figure 4 – Antibiotic susceptibility using Etest® strips. (p. 11)

Supplementary Table 1 - Summary of mutations. Here we highlight all mutations found in both normal gravity and simulated microgravity populations of which had a frequency of mutation greater than 0.1. Frequencies colored in blue indicate mutations in which the variant was unique to the normal gravity environment, frequencies colored in orange indicate the variants that were shared between both normal gravity and simulated microgravity populations at the frequencies colored in green indicate variants that are unique to the simulated microgravity environment. Frequencies highlighted in yellow are selective sweeps (f = 1.000).

Time Point	Position	Mutation	NG1	NG2	NG3	NG4	MG1	MG2	MG3	MG4	Annotation	Gene	Homologous SMU gene	Description
			Frequency of mutation for each population											
Ancestral	702,753	T→G	1.000	1.000	1.000	1.000	1.000	1.000	1.000	1.000	I365S (ATT→AGT)	*DQM59_RS03765* →		UDP-N-acetylglucosamine 2-epimerase (non-hydrolyzing)
	726,961:1	+T	1.000	1.000	1.000	1.000	1.000	1.000	1.000	1.000	pseudogene (316/659 nt)	*DQM59_RS03875* →		TIGR01906 family membrane protein
	1,622,529:1	+T	1.000	1.000	1.000	1.000	1.000	1.000	1.000	1.000	intergenic (-15/+47)	*trxB* ← / ← *DQM59_RS08210*		thioredoxin-disulfide reductase/DUF4059 family protein
21-days	1,988,512	A→C			0.108	0.237					F52V (TTT→GTT)	*DQM59_RS10195* ←	SMU_2137c	hypothetical protein
	1,988,605	G→T			0.265	0.178					H21N (CAT→AAT)	*DQM59_RS10195* ←	SMU_2137c	hypothetical protein
	835,787	C→A	0.076	0.140							L168I (CTC→ATC)	*DQM59_RS04335* →	SMU_1307c	DUF1003 domain-containing protein
	716,798	+AATATAG			0.264			0.285			intergenic (+159/-70)	*DQM59_RS03820* → / → *lpdA*	SMU_130	AAA domain-containing protein/dihydrolipoyl dehydrogenase
	716,816	G→T	0.212	0.190	0.146	0.162	0.121	0.412			intergenic (+177/-52)	*DQM59_RS03820* → / → *lpdA*	SMU_130	AAA domain-containing protein/dihydrolipoyl dehydrogenase
	716,817	C→A	0.240	0.120	0.087	0.052	0.324	0.185	0.259	0.339	intergenic (+178/-51)	*DQM59_RS03820* → / → *lpdA*	SMU_130	AAA domain-containing protein/dihydrolipoyl dehydrogenase
	716,818	G→T	0.053				0.059				intergenic (+179/-50)	*DQM59_RS03820* → / → *lpdA*	SMU_130	AAA domain-containing protein/dihydrolipoyl dehydrogenase
	716,823	C→T					0.152				intergenic (+184/-45)	*DQM59_RS03820* → / → *lpdA*	SMU_130	AAA domain-containing protein/dihydrolipoyl dehydrogenase
	1,677,849	G→T					0.101				S112Y (TCT→TAT)	*DQM59_RS08505* ←	SMU_399	hypothetical protein (C3-glycoprotein degrading protease)
	1,677,871	Δ12 bp						0.063	0.262		coding (302-313/759 nt)	*DQM59_RS08505* ←	SMU_399	hypothetical protein (C3-glycoprotein degrading protease)
	1,678,012	C→A					0.104				E58* (GAG→TAG)	*DQM59_RS08505* ←	SMU_399	hypothetical protein (C3-glycoprotein degrading protease)
	1,678,064	+GAATTCTCT TCC								0.060	coding (120/759 nt)	*DQM59_RS08505* ←	SMU_399	hypothetical protein (C3-glycoprotein degrading protease)
	1,678,148	Δ13 bp								0.135	coding (24-36/759 nt)	*DQM59_RS08505* ←	SMU_399	hypothetical protein (C3-glycoprotein degrading protease)
	357,172	T→A					0.054				F331L (TTT→TTA)	*DQM59_RS01880* →	SMU_1830	Cof-type HAD-IIB family hydrolase
	1,838,520	2 bp→11 bp					0.333				pseudogene (409-410/859 nt)	*DQM59_RS09330* →		IS982 family transposase
42-days	1,988,566	G→T	0.077								R34S (CGT→AGT)	*DQM59_RS10195* ←	SMU_2137c	hypothetical protein
	1,988,452	C→T		0.051							E72K (GAA→AAA)	*DQM59_RS10195* ←	SMU_2137c	hypothetical protein
	1,988,621	T→G		0.240							K15N (AAA→AAC)	*DQM59_RS10195* ←	SMU_2137c	hypothetical protein
	1,988,512	A→C			0.076	0.170					F52V (TTT→GTT)	*DQM59_RS10195* ←	SMU_2137c	hypothetical protein
	1,988,605	G→T			0.117	0.134					H21N (CAT→AAT)	*DQM59_RS10195* ←	SMU_2137c	hypothetical protein
	834,868	C→T			0.381	0.138					Q31* (CAG→TAG)	*DQM59_RS04330* →	N/A	RidA family protein
	1,318,182	A→C	0.077	0.179							F219L (TTT→TTG)	*DQM59_RS06715* ←	SMU_778	shikimate dehydrogenase
	1,535,722	C→A	0.353								K373N (AAG→AAT)	*DQM59_RS07770* ←	SMU_548	UDP-N-acetylmuramoyl-L-alanine--D-glutamate ligase
	1,600,333	C→A			0.054						G174C (GGT→TGT)	*pknB* ←	SMU_484	Stk1 family PASTA domain-containing Ser/Thr kinase
	1,600,720	G→A			0.132						R45C (CGT→TGT)	*pknB* ←	SMU_484	Stk1 family PASTA domain-containing Ser/Thr kinase
	1,599,428	G→C				0.118					Y475* (TAC→TAG)	*pknB* ←	SMU_484	Stk1 family PASTA domain-containing Ser/Thr kinase
	1,477,393	Δ81 bp	0.744	0.151							coding (2051-2131/4689 nt)	*spaP* ←	SMU_610	cell surface antigen I/II
	1,477,362	G→A				0.077					S721L (TCA→TTA)	*spaP* ←	SMU_610	cell surface antigen I/II
	507,736	A→T	0.053								intergenic (-76/-66)	*tehB* ← / → *DQM59_RS02795*		SAM-dependent methyltransferase TehB/hypothetical protein
	619,952	A→G		0.101							T104A (ACA→GCA)	*vicK* →	SMU_1516	cell wall metabolism sensor histidine kinase VicK
	348,924	T→C		0.065							V31A (GTA→GCA)	*DQM59_RS01845* →	SMU_1836	3-deoxy-7-phosphoheptulonate synthase
	349,072	A→C			0.185						I80I (ATA→ATC)	*DQM59_RS01845* →	SMU_1836	3-deoxy-7-phosphoheptulonate synthase
	349,108	C→T			0.106						R92R (CGC→CGT)	*DQM59_RS01845* →	SMU_1836	3-deoxy-7-phosphoheptulonate synthase
	348,924	T→C				0.168					V31A (GTA→GCA)	*DQM59_RS01845* →	SMU_1836	3-deoxy-7-phosphoheptulonate synthase
	349,072	A→C				0.172					I80I (ATA→ATC)	*DQM59_RS01845* →	SMU_1836	3-deoxy-7-phosphoheptulonate synthase
	349,957	C→T				0.060					A31V (GCA→GTA)	*DQM59_RS01850* →	SMU_1836	3-deoxy-7-phosphoheptulonate synthase
	350,105	C→A					0.121				I80I (ATC→ATA)	*DQM59_RS01850* →	SMU_1836	3-deoxy-7-phosphoheptulonate synthase
	350,141	T→C					0.134				R92R (CGT→CGC)	*DQM59_RS01850* →	SMU_1836	3-deoxy-7-phosphoheptulonate synthase
	716,816	G→T	0.167	0.521	0.254	0.218	0.248	0.221	0.056		intergenic (+177/-52)	*DQM59_RS03820* → / → *lpdA*	SMU_130	AAA domain-containing protein/dihydrolipoyl dehydrogenase
	716,817	C→A				0.141	0.201	0.124			intergenic (+178/-51)	*DQM59_RS03820* → / → *lpdA*	SMU_130	AAA domain-containing protein/dihydrolipoyl dehydrogenase
	716,688	G→T					0.497				intergenic (+49/-180)	*DQM59_RS03820* → / → *lpdA*	SMU_130	AAA domain-containing protein/dihydrolipoyl dehydrogenase
	1,677,670	Δ239 bp	0.291								coding (276-514/759 nt)	*DQM59_RS08505* ←	SMU_399	hypothetical protein (C3-glycoprotein degrading protease)
	1,677,871	Δ12 bp	0.089					0.580	0.882		coding (302-313/759 nt)	*DQM59_RS08505* ←	SMU_399	hypothetical protein (C3-glycoprotein degrading protease)
	1,677,643	Δ10 bp					0.076				coding (532-541/759 nt)	*DQM59_RS08505* ←	SMU_399	hypothetical protein (C3-glycoprotein degrading protease)
	1,677,849	G→T					0.473				S112Y (TCT→TAT)	*DQM59_RS08505* ←	SMU_399	hypothetical protein (C3-glycoprotein degrading protease)
	1,678,012	C→A					0.094				E58* (GAG→TAG)	*DQM59_RS08505* ←	SMU_399	hypothetical protein (C3-glycoprotein degrading protease)
	1,677,523	C→A						0.102			E221* (GAA→TAA)	*DQM59_RS08505* ←	SMU_399	hypothetical protein (C3-glycoprotein degrading protease)

	1,677,646	(CCATGACTC TCAACAAAAT CTAA)$_{1\rightarrow 2}$								0.575	coding (538/759 nt)	*DQM59_RS08505* ←	SMU_399	hypothetical protein (C3-glycoprotein degrading protease)
	1,060,839	G→A					0.129				Q202* (CAA→TAA)	*DQM59_RS05510* ←	SMU_1053	redox-sensing transcriptional repressor Rex
	1,060,979	G→T					0.056				T155K (ACA→AAA)	*DQM59_RS05510* ←	SMU_1053	redox-sensing transcriptional repressor Rex
	1,061,250	C→T					0.065				G65S (GGT→AGT)	*DQM59_RS05510* ←	SMU_1053	redox-sensing transcriptional repressor Rex
	1,061,289	C→T					0.053				D52N (GAT→AAT)	*DQM59_RS05510* ←	SMU_1053	redox-sensing transcriptional repressor Rex
	1,061,291	C→A					0.065				R51L (CGT→CTT)	*DQM59_RS05510* ←	SMU_1053	redox-sensing transcriptional repressor Rex
	1,061,306	G→A					0.066				S46L (TCA→TTA)	*DQM59_RS05510* ←	SMU_1053	redox-sensing transcriptional repressor Rex
	1,061,246	T→C						0.224			Y66C (TAT→TGT)	*DQM59_RS05510* ←	SMU_1053	redox-sensing transcriptional repressor Rex
	1,060,865	T→G							0.833		Q193P (CAA→CCA)	*DQM59_RS05510* ←	SMU_1053	redox-sensing transcriptional repressor Rex
	1,061,345	G→T								0.656	A33E (GCA→GAA)	*DQM59_RS05510* ←	SMU_1053	redox-sensing transcriptional repressor Rex
	1,411,252	C→G					0.194	0.495			G54A (GGT→GCT)	*DQM59_RS07180* ←	SMU_674	phosphocarrier protein HPr
63-days	129,860	163 bp x 2		0.426							duplication	[*DQM59_RS00685*]	SMU_127	[*DQM59_RS00685*]
	129,830	Δ16 bp			0.056						intergenic (+284/-77)	*DQM59_RS00680* → / → *DQM59 _RS00685*	SMU_127	NAD(P)H-dependent oxidoreductase/thiamine pyrophosphate-dependent dehydrogenase E1 component subunit alpha
	129,826	G→A				0.234					intergenic (+280/-96)	*DQM59_RS00680* → / → *DQM59 _RS00685*	SMU_127	NAD(P)H-dependent oxidoreductase/thiamine pyrophosphate-dependent dehydrogenase E1 component subunit alpha
	349,222	A→G	0.085								E130E (GAA→GAG)	*DQM59_RS01845* →	SMU_1836	3-deoxy-7-phosphoheptulonate synthase
	349,225	G→A	0.076								T131T (ACG→ACA)	*DQM59_RS01845* →	SMU_1836	3-deoxy-7-phosphoheptulonate synthase
	349,228	T→A	0.073								G132G (GGT→GGA)	*DQM59_RS01845* →	SMU_1836	3-deoxy-7-phosphoheptulonate synthase
	349,229	A→T	0.073								I133F (ATT→TTT) ‡	*DQM59_RS01845* →	SMU_1836	3-deoxy-7-phosphoheptulonate synthase
	349,231	T→G	0.067								I133M (ATT→ATG) ‡	*DQM59_RS01845* →	SMU_1836	3-deoxy-7-phosphoheptulonate synthase
	349,252	C→T	0.070								L140L (CTC→CTT)	*DQM59_RS01845* →	SMU_1836	3-deoxy-7-phosphoheptulonate synthase
	349,255	T→C	0.054								Y141Y (TAT→TAC)	*DQM59_RS01845* →	SMU_1836	3-deoxy-7-phosphoheptulonate synthase
	348,924	T→C		0.054							V31A (GTA→GCA)	*DQM59_RS01845* →	SMU_1836	3-deoxy-7-phosphoheptulonate synthase
	349,072	A→C			0.143						I80I (ATA→ATC)	*DQM59_RS01845* →	SMU_1836	3-deoxy-7-phosphoheptulonate synthase
	349,108	C→T			0.104						R92R (CGC→CGT)	*DQM59_RS01845* →	SMU_1836	3-deoxy-7-phosphoheptulonate synthase
	349,072	A→C				0.195					I80I (ATA→ATC)	*DQM59_RS01845* →	SMU_1836	3-deoxy-7-phosphoheptulonate synthase
	349,108	C→T				0.124					R92R (CGC→CGT)	*DQM59_RS01845* →	SMU_1836	3-deoxy-7-phosphoheptulonate synthase
	350,210	T→C	0.071								N115N (AAT→AAC)	*DQM59_RS01850* →	SMU_1836	3-deoxy-7-phosphoheptulonate synthase
	350,213	C→T	0.072								G116G (GGC→GGT)	*DQM59_RS01850* →	SMU_1836	3-deoxy-7-phosphoheptulonate synthase
	350,217	C→A	0.071								H118N (CAT→AAT) ‡	*DQM59_RS01850* →	SMU_1836	3-deoxy-7-phosphoheptulonate synthase
	350,218	A→C	0.069								H118P (CAT→CCT) ‡	*DQM59_RS01850* →	SMU_1836	3-deoxy-7-phosphoheptulonate synthase
	350,219	T→A	0.069								H118Q (CAT→CAA) ‡	*DQM59_RS01850* →	SMU_1836	3-deoxy-7-phosphoheptulonate synthase
	350,225	T→A	0.087								V120V (GTT→GTA)	*DQM59_RS01850* →	SMU_1836	3-deoxy-7-phosphoheptulonate synthase
	350,228	T→C	0.087								R121R (CGT→CGC)	*DQM59_RS01850* →	SMU_1836	3-deoxy-7-phosphoheptulonate synthase
	350,229	A→C	0.088								N122H (AAT→CAT)	*DQM59_RS01850* →	SMU_1836	3-deoxy-7-phosphoheptulonate synthase
	350,232	T→C	0.087								L123L (TTG→CTG)	*DQM59_RS01850* →	SMU_1836	3-deoxy-7-phosphoheptulonate synthase
	350,105	C→A		0.061							I80I (ATC→ATA)	*DQM59_RS01850* →	SMU_1836	3-deoxy-7-phosphoheptulonate synthase
	349,957	C→T				0.090					A31V (GCA→GTA)	*DQM59_RS01850* →	SMU_1836	3-deoxy-7-phosphoheptulonate synthase
	406,325	C→T			0.103						intergenic (+48/-47)	*DQM59_RS02185* → / → *cas5c*	SMU_1763c	CRISPR-associated helicase/endonuclease Cas3/type I-C CRISPR-associated protein Cas5
	450,068	A→C	0.223								K7Q (AAA→CAA)	*DQM59_RS02455* →	SMU_1715c	CBS domain-containing protein
	619,555	C→T		0.205							R205C (CGT→TGT)	*DQM59_RS03385* →	N/A	response regulator transcription factor
	619,555	C→T				0.053					R205C (CGT→TGT)	*DQM59_RS03385* →	N/A	response regulator transcription factor
	834,868	C→T			0.284	0.460					Q31* (CAG→TAG)	*DQM59_RS04330* →	N/A	RidA family protein
	1,022,856	G→T	0.233								L404I (CTT→ATT)	*DQM59_RS05315* ←	SMU_1093	ABC transporter permease
	1,318,182	A→C		0.711							F219L (TTT→TTG)	*DQM59_RS06715* ←	SMU_778	shikimate dehydrogenase
	1,535,722	C→A	0.224								K373N (AAG→AAT)	*DQM59_RS07770* ←	SMU_548	UDP-N-acetylmuramoyl-L-alanine--D-glutamate ligase
	1,709,058	C→A		0.161							G427* (GGA→TGA)	*gltB* ←		glutamate synthase large subunit
	1,477,393	Δ81 bp	0.592	0.052							coding (2051-2131/4689 nt)	*spaP* ←	SMU_610	cell surface antigen I/II
	1,477,362	G→A			0.082						S721L (TCA→TTA)	*spaP* ←	SMU_610	cell surface antigen I/II
	1,476,773	A→G				0.086					N917N (AAT→AAC)	*spaP* ←	SMU_610	cell surface antigen I/II

	236,958	T→G			0.181						F30V (TTT→GTT)	*DQM59_RS01220* →		DUF1033 family protein
	237,078	C→T							0.336		Q70* (CAA→TAA)	*DQM59_RS01220* →		DUF1033 family protein
	716,816	G→T	0.206	0.327	0.089				0.164		intergenic (+177/-52)	*DQM59_RS03820* → / → *lpdA*	SMU_130	AAA domain-containing protein/dihydrolipoyl dehydrogenase
	716,688	G→T					1.000				intergenic (+49/-180)	*DQM59_RS03820* → / → *lpdA*	SMU_130	AAA domain-containing protein/dihydrolipoyl dehydrogenase
	716,817	C→A						0.066			intergenic (+178/-51)	*DQM59_RS03820* → / → *lpdA*	SMU_130	AAA domain-containing protein/dihydrolipoyl dehydrogenase
	835,826	C→T	0.071								R181C (CGT→TGT)	*DQM59_RS04335* →	SMU_1307c	DUF1003 domain-containing protein
	835,978	C→A	0.100								S231R (AGC→AGA)	*DQM59_RS04335* →	SMU_1307c	DUF1003 domain-containing protein
	835,755	C→T								1	A157V (GCT→GTT)	*DQM59_RS04335* →	SMU_1307c	DUF1003 domain-containing protein
	1,061,274	C→T					0.075				G57S (GGC→AGC)	*DQM59_RS05510* ←	SMU_1053	redox-sensing transcriptional repressor Rex
	1,061,345	G→T					0.365				A33E (GCA→GAA)	*DQM59_RS05510* ←	SMU_1053	redox-sensing transcriptional repressor Rex
	1,061,291	C→T	0.245								R51H (CGT→CAT)	*DQM59_RS05510* ←	SMU_1053	redox-sensing transcriptional repressor Rex
	1,061,402	C→T		0.073							R14H (CGT→CAT)	*DQM59_RS05510* ←	SMU_1053	redox-sensing transcriptional repressor Rex
	1,060,979	G→C			0.256						T155R (ACA→AGA)	*DQM59_RS05510* ←	SMU_1053	redox-sensing transcriptional repressor Rex
	1,061,300	G→A			0.607						T48I (ACA→ATA)	*DQM59_RS05510* ←	SMU_1053	redox-sensing transcriptional repressor Rex
	1,061,265	C→T				0.051					G60S (GGC→AGC)	*DQM59_RS05510* ←	SMU_1053	redox-sensing transcriptional repressor Rex
	1,061,303	G→A				0.095					A47V (GCG→GTG)	*DQM59_RS05510* ←	SMU_1053	redox-sensing transcriptional repressor Rex
	1,061,246	T→C						0.128			Y66C (TAT→TGT)	*DQM59_RS05510* ←	SMU_1053	redox-sensing transcriptional repressor Rex
	1,060,865	T→G							0.525		Q193P (CAA→CCA)	*DQM59_RS05510* ←	SMU_1053	redox-sensing transcriptional repressor Rex
	1,061,099	G→T							0.052		A115E (GCA→GAA)	*DQM59_RS05510* ←	SMU_1053	redox-sensing transcriptional repressor Rex
	1,411,252	C→A		0.327		0.205	0.433	0.720	0.338	1	G54V (GGT→GTT)	*DQM59_RS07180* ←	SMU_674	phosphocarrier protein HPr
	1,677,670	Δ239 bp	0.298								coding (276-514/759 nt)	*DQM59_RS08505* ←	SMU_399	hypothetical protein (C3-glycoprotein degrading protease)
	1,677,871	Δ12 bp	0.081							1	coding (302-313/759 nt)	*DQM59_RS08505* ←	SMU_399	hypothetical protein (C3-glycoprotein degrading protease)
	1,677,849	G→T					1.000				S112Y (TCT→TAT)	*DQM59_RS08505* ←	SMU_399	hypothetical protein (C3-glycoprotein degrading protease)
	1,677,796	G→A			0.108						Q130* (CAA→TAA)	*DQM59_RS08505* ←	SMU_399	hypothetical protein (C3-glycoprotein degrading protease)
	1,677,871	Δ12 bp						0.847	0.634		coding (302-313/759 nt)	*DQM59_RS08505* ←	SMU_399	hypothetical protein (C3-glycoprotein degrading protease)
	1,677,520	+TTCAAGATA ATTCT							0.093		coding (664/759 nt)	*DQM59_RS08505* ←	SMU_399	hypothetical protein (C3-glycoprotein degrading protease)
	1,678,155	+AAAAC							0.225		coding (29/759 nt)	*DQM59_RS08505* ←	SMU_399	hypothetical protein (C3-glycoprotein degrading protease)
	1,988,566	G→T	0.251								R34S (CGT→AGT)	*DQM59_RS10195* ←	SMU_2137c	hypothetical protein
	1,988,605	G→A	0.080								H21Y (CAT→TAT)	*DQM59_RS10195* ←	SMU_2137c	hypothetical protein
	1,988,621	T→G		0.541							K15N (AAA→AAC)	*DQM59_RS10195* ←	SMU_2137c	hypothetical protein
	1,988,510	A→C			0.579						F52L (TTT→TTG) ‡	*DQM59_RS10195* ←	SMU_2137c	hypothetical protein
	1,988,512	A→C			0.095						F52V (TTT→GTT) ‡	*DQM59_RS10195* ←	SMU_2137c	hypothetical protein
	1,988,605	G→T				0.355					H21N (CAT→AAT)	*DQM59_RS10195* ←	SMU_2137c	hypothetical protein
	1,988,501	T→G						0.122			E55D (GAA→GAC)	*DQM59_RS10195* ←	SMU_2137c	hypothetical protein
	1,599,170	A→C	0.349								Y561* (TAT→TAG)	*pknB* ←	SMU_484	Stk1 family PASTA domain-containing Ser/Thr kinase
	1,600,606	C→G	0.116								D83H (GAC→CAC)	*pknB* ←	SMU_484	Stk1 family PASTA domain-containing Ser/Thr kinase
	1,600,720	G→A			0.285	0.269					R45C (CGT→TGT)	*pknB* ←	SMU_484	Stk1 family PASTA domain-containing Ser/Thr kinase
	1,600,619	G→T						0.084			D78E (GAC→GAA)	*pknB* ←	SMU_484	Stk1 family PASTA domain-containing Ser/Thr kinase
	1,600,081	G→A								0.862	R258C (CGC→TGC)	*pknB* ←	SMU_484	Stk1 family PASTA domain-containing Ser/Thr kinase
	1,227,749	A→C					0.115				Y636* (TAT→TAG)	*DQM59_RS06275* ←		PTS fructose transporter subunit IIABC
	233,349	G→C					1.000				G88A (GGT→GCT)	*rpoC* →		DNA-directed RNA polymerase subunit beta'
	548,031	G→T						0.204			G123C (GGT→TGT)	*ccpA* →	SMU_1591	catabolite control protein A
	882,760	C→T						0.123			A78V (GCA→GTA)	*DQM59_RS04610* →	N/A	phosphopyruvate hydratase
	1,114,760	G→T							0.113		L22I (CTC→ATC)	*ylqF* ←	N/A	ribosome biogenesis GTPase YlqF
100-days	129,860	163 bp x 2	0.105								duplication	*[DQM59_RS00685]*	SMU_127	*[DQM59_RS00685]*
	1,418,251	Δ1 bp		1.000							intergenic (-236/-20)	*acnA* ← / → *DQM59_RS07205*	SMU_670/SMU_669c	aconitate hydratase AcnA/redoxin NrdH
	129,826	G→A				0.333					intergenic (+280/-96)	*DQM59_RS00680* → / → *DQM59_RS00685*	SMU_127	NAD(P)H-dependent oxidoreductase/thiamine pyrophosphate-dependent dehydrogenase E1 component subunit alpha
	149,107	G→T				0.102					F162L (TTC→TTA)	*DQM59_RS00765* ←	SMU_144c	cyclic nucleotide-binding domain-containing protein
	255,767	Δ30 bp		0.619							coding (464-493/495 nt)	*DQM59_RS01355* →	SMU_1185	PTS sugar transporter subunit IIB
	349,072	A→C				0.360					I80I (ATA→ATC)	*DQM59_RS01845* →	SMU_1836	3-deoxy-7-phosphoheptulonate synthase
	349,108	C→T				0.203					R92R (CGC→CGT)	*DQM59_RS01845* →	SMU_1836	3-deoxy-7-phosphoheptulonate synthase
	581,370	C→T		0.925							P169L (CCT→CTT)	*DQM59_RS03185* →	SMU_1708	potassium uptake protein TrkA

582,843 C→T			0.195						intergenic (-151/-338)	*DQM59_RS03195* ← / → *DQM59_RS03200*	N/A	EamA family transporter/GNAT family N-acetyltransferase
584,019 T→G			0.527						F98V (TTT→GTT)	*DQM59_RS03205* →	N/A	CBS domain-containing protein
792,070 C→T				0.170					Q1557* (CAA→TAA)	*DQM59_RS04175* →	SMU_1142c	non-ribosomal peptide synthetase
834,868 C→T				0.547					Q31* (CAG→TAG)	*DQM59_RS04330* →	N/A	RidA family protein
953,468 C→A				0.172					intergenic (+95/-2)	*DQM59_RS04955* → / → *DQM59_RS04960*	N/A	cyclase family protein/HAD-IIB family hydrolase
977,684 C→A				0.102					intergenic (+1/-60)	*DQM59_RS05085* → / → *DQM59_RS05090*	SMU_1142c	bifunctional riboflavin kinase/FAD synthetase/transcriptional regulator Spx
1,323,448 G→A			0.331						P239S (CCT→TCT)	*DQM59_RS06735* ←	N/A	HAD family hydrolase
1,508,460 C→A				0.181					S191* (TCG→TAG)	*DQM59_RS07640* →	SMU_577	sensor protein LytS
1,508,573 C→T				0.150					Q229* (CAG→TAG)	*DQM59_RS07640* →	SMU_577	sensor protein LytS
1,838,656 G→T				0.187					pseudogene (545/859 nt)	*DQM59_RS09330* →		IS982 family transposase
426,926 C→A				0.114					L140I (CTC→ATC)	*fabD* →	SMU_1741	ACP S-malonyltransferase
1,514,225 G→C				0.148					A648G (GCT→GGT)	*feoB* ←	SMU_570	ferrous iron transport protein B
686,441 G→T				0.227			0.556		E138D (GAG→GAT)	*DQM59_RS03695* →	SMU_1453c	hypothetical protein
716,816 G→T	0.920		0.821	0.247	0.120	0.691	0.941		intergenic (+177/-52)	*DQM59_RS03820* → / → *lpdA*	SMU_130	AAA domain-containing protein/dihydrolipoyl dehydrogenase
716,823 C→T				0.130					intergenic (+184/-45)	*DQM59_RS03820* → / → *lpdA*	SMU_130	AAA domain-containing protein/dihydrolipoyl dehydrogenase
716,688 G→T					0.876				intergenic (+49/-180)	*DQM59_RS03820* → / → *lpdA*	SMU_130	AAA domain-containing protein/dihydrolipoyl dehydrogenase
716,817 C→A					0.169				intergenic (+178/-51)	*DQM59_RS03820* → / → *lpdA*	SMU_130	AAA domain-containing protein/dihydrolipoyl dehydrogenase
835,869 A→G	0.656								E195G (GAA→GGA)	*DQM59_RS04335* →	SMU_1307c	DUF1003 domain-containing protein
835,880 C→T	0.177								R199C (CGT→TGT)	*DQM59_RS04335* →	SMU_1307c	DUF1003 domain-containing protein
835,826 C→T		0.930							R181C (CGT→TGT)	*DQM59_RS04335* →	SMU_1307c	DUF1003 domain-containing protein
835,978 C→A		0.948							S231R (AGC→AGA)	*DQM59_RS04335* →	SMU_1307c	DUF1003 domain-containing protein
835,583 C→T			0.289						Q100* (CAG→TAG)	*DQM59_RS04335* →	SMU_1307c	DUF1003 domain-containing protein
835,703 C→T			0.542						R140* (CGA→TGA)	*DQM59_RS04335* →	SMU_1307c	DUF1003 domain-containing protein
835,573 T→A				0.242					D96E (GAT→GAA)	*DQM59_RS04335* →	SMU_1307c	DUF1003 domain-containing protein
835,589 A→C					0.126				K102Q (AAG→CAG)	*DQM59_RS04335* →	SMU_1307c	DUF1003 domain-containing protein
835,989 C→G					0.478				T235R (ACA→AGA)	*DQM59_RS04335* →	SMU_1307c	DUF1003 domain-containing protein
835,787 C→A						0.113			L168I (CTC→ATC)	*DQM59_RS04335* →	SMU_1307c	DUF1003 domain-containing protein
835,573 T→A							0.527		D96E (GAT→GAA)	*DQM59_RS04335* →	SMU_1307c	DUF1003 domain-containing protein
835,781 G→T							0.110		A166S (GCT→TCT) ‡	*DQM59_RS04335* →	SMU_1307c	DUF1003 domain-containing protein
835,782 C→A							0.137		A166D (GCT→GAT) ‡	*DQM59_RS04335* →	SMU_1307c	DUF1003 domain-containing protein
835,755 C→T								0.929	A157V (GCT→GTT)	*DQM59_RS04335* →	SMU_1307c	DUF1003 domain-containing protein
1,003,557 C→G				0.087					G179A (GGT→GCT)	*DQM59_RS05220* ←	SMU_1115	L-lactate dehydrogenase
1,003,666 C→A				0.150					A143S (GCC→TCC)	*DQM59_RS05220* ←	SMU_1115	L-lactate dehydrogenase
1,003,918 C→G					0.805				A59P (GCT→CCT)	*DQM59_RS05220* ←	SMU_1115	L-lactate dehydrogenase
1,061,291 C→A				0.198					R51L (CGT→CTT)	*DQM59_RS05510* ←	SMU_1053	redox-sensing transcriptional repressor Rex
1,061,280 A→C						0.582			Y55D (TAT→GAT)	*DQM59_RS05510* ←	SMU_1053	redox-sensing transcriptional repressor Rex
1,352,053 C→A			0.903						E244* (GAA→TAA)	*DQM59_RS06875* ←	SMU_1830	Cof-type HAD-IIB family hydrolase
1,352,058 G→T				0.256					A242E (GCA→GAA)	*DQM59_RS06875* ←	SMU_1830	Cof-type HAD-IIB family hydrolase
1,352,622 C→G				0.154					G54A (GGC→GCC)	*DQM59_RS06875* ←	SMU_1830	Cof-type HAD-IIB family hydrolase
1,352,058 G→T							0.531		A242E (GCA→GAA)	*DQM59_RS06875* ←	SMU_1830	Cof-type HAD-IIB family hydrolase
1,411,252 C→A				0.063	0.061			0.933	G54V (GGT→GTT)	*DQM59_RS07180* ←	SMU_674	phosphocarrier protein HPr
1,426,369 C→A				0.237			0.483		A180S (GCA→TCA)	*DQM59_RS07235* ←	SMU_663	N-acetyl-gamma-glutamyl-phosphate reductase
1,509,927 G→T				0.067					E106* (GAG→TAG)	*DQM59_RS07645* →	SMU_576	response regulator
1,510,302 G→A				0.150					G231R (GGG→AGG)	*DQM59_RS07645* →	SMU_576	response regulator
1,510,339 C→A							0.216		T243N (ACC→AAC)	*DQM59_RS07645* →	SMU_576	response regulator
1,677,967:1 +T	1.000		1.000			1.000			coding (217/759 nt)	*DQM59_RS08505* ←	SMU_399	hypothetical protein (C3-glycoprotein degrading protease)
1,677,871 Δ12 bp		1.000		0.257		0.174	0.655	1.000	coding (302-313/759 nt)	*DQM59_RS08505* ←	SMU_399	hypothetical protein (C3-glycoprotein degrading protease)
1,677,849 G→T					0.864				S112Y (TCT→TAT)	*DQM59_RS08505* ←	SMU_399	hypothetical protein (C3-glycoprotein degrading protease)
1,678,012 C→A					0.104				E58* (GAG→TAG)	*DQM59_RS08505* ←	SMU_399	hypothetical protein (C3-glycoprotein degrading protease)
1,677,523 C→A						0.090			E221* (GAA→TAA)	*DQM59_RS08505* ←	SMU_399	hypothetical protein (C3-glycoprotein degrading protease)
1,678,148 Δ13 bp								0.059	coding (24-36/759 nt)	*DQM59_RS08505* ←	SMU_399	hypothetical protein (C3-glycoprotein degrading protease)
1,988,621 T→G	0.108								K15N (AAA→AAC)	*DQM59_RS10195* ←	SMU_2137c	hypothetical protein
1,988,512 A→C			0.056						F52V (TTT→GTT)	*DQM59_RS10195* ←	SMU_2137c	hypothetical protein

1,988,605 G→T				0.654			H21N (CAT→AAT)	*DQM59_RS10195* ←	SMU_2137c	hypothetical protein
1,988,403 T→C					0.370		Y88C (TAT→TGT)	*DQM59_RS10195* ←	SMU_2137c	hypothetical protein
440,770 C→A		0.234					F432L (TTC→TTA)	*mltG* →	N/A	endolytic transglycosylase MltG
440,770 C→A					0.560		F432L (TTC→TTA)	*mltG* →	N/A	endolytic transglycosylase MltG
1,600,680 A→G		0.133					F58S (TTT→TCT)	*pknB* ←	SMU_484	Stk1 family PASTA domain-containing Ser/Thr kinase
1,600,720 G→A		0.359					R45C (CGT→TGT)	*pknB* ←	SMU_484	Stk1 family PASTA domain-containing Ser/Thr kinase
1,600,848 A→C			0.160				I2S (ATT→AGT)	*pknB* ←	SMU_484	Stk1 family PASTA domain-containing Ser/Thr kinase
1,600,081 G→A						0.093	R258C (CGC→TGC)	*pknB* ←	SMU_484	Stk1 family PASTA domain-containing Ser/Thr kinase
1,600,797 C→G						0.600	G19A (GGA→GCA)	*pknB* ←	SMU_484	Stk1 family PASTA domain-containing Ser/Thr kinase
1,477,362 G→A		0.204					S721L (TCA→TTA)	*spaP* ←	SMU_610	cell surface antigen I/II
1,477,823 Δ114 bp					0.190		coding (1588-1701/4689 nt)	*spaP* ←	SMU_610	cell surface antigen I/II
619,883 G→T	0.595						V81F (GTT→TTT)	*vicK* →	SMU_1516	cell wall metabolism sensor histidine kinase VicK
620,861 A→T	0.376						I407F (ATT→TTT)	*vicK* →	SMU_1516	cell wall metabolism sensor histidine kinase VicK
620,352 C→A		0.258					A237D (GCC→GAC)	*vicK* →	SMU_1516	cell wall metabolism sensor histidine kinase VicK
620,971 G→A				0.154			W443* (TGG→TGA)	*vicK* →	SMU_1516	cell wall metabolism sensor histidine kinase VicK
620,352 C→A					0.409		A237D (GCC→GAC)	*vicK* →	SMU_1516	cell wall metabolism sensor histidine kinase VicK
833,322 G→T						0.141	intergenic (+181/+120)	*asnS* → / ← *DQM59_RS04320*	SMU_1311	asparagine--tRNA ligase/hypothetical protein
67,850 G→C				0.199			G77R (GGT→CGT)	*DQM59_RS00400* →	SMU_66	membrane protein
97,695 C→A			0.836				intergenic (-381/-244)	*DQM59_RS00535* ← / → *DQM59_RS00540*	SMU_96	IS982 family transposase/DNA-directed RNA polymerase subunit delta
581,818 T→G					0.149		V96G (GTT→GGT)	*DQM59_RS03190* →	SMU_1561	potassium transporter TrkB
749,929 A→C					0.149		D80A (GAT→GCT)	*DQM59_RS03980* →	SMU_1394	hypothetical protein
749,933 A→C					0.150		L81F (TTA→TTC)	*DQM59_RS03980* →	SMU_1394	hypothetical protein
1,023,267 T→G				0.130			M267L (ATG→CTG)	*DQM59_RS05315* ←	SMU_1093	ABC transporter permease
1,728,360 G→T					0.315		pseudogene (217/304 nt)	*DQM59_RS08775* ←	SMU_1232c	DUF1697 domain-containing protein
1,260,188 C→A				0.114			E251* (GAG→TAG)	*gorA* ←	SMU_838	glutathione-disulfide reductase
233,349 G→C			0.877				G88A (GGT→GCT)	*rpoC* →	SMU_1989	DNA-directed RNA polymerase subunit beta'

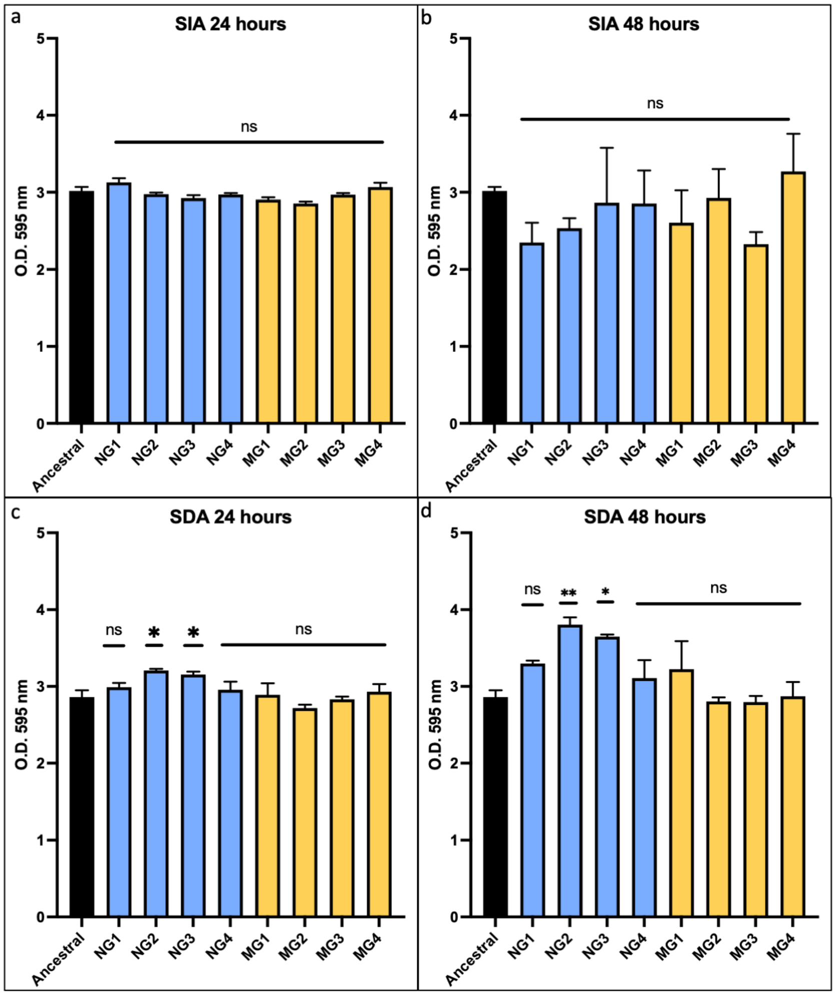

Supplementary Figure 1 – Adhesion after 21-days shows little change. We assessed both SIA after A) 24- and B) 48-hours and SIA for both C) 24- and D) 48-hours for the normal gravity and simulated microgravity populations after 21-days of adaptation. Data shows that there is almost no change in these phenotypes when compared to the ancestral populations. Data was plotted in GraphPad Prism ® 9.2.0 and unpaired t-tests with 95% confidence were used to calculated significant differences for pairwise comparisons between the ancestral and each treatment populations. Error bars are standard error means and significance from T-tests are reported as a two-tailed p-value where * ≤ 0.05, ** ≤ 0.01, *** ≤ 0.001 and **** ≤ 0.0001.

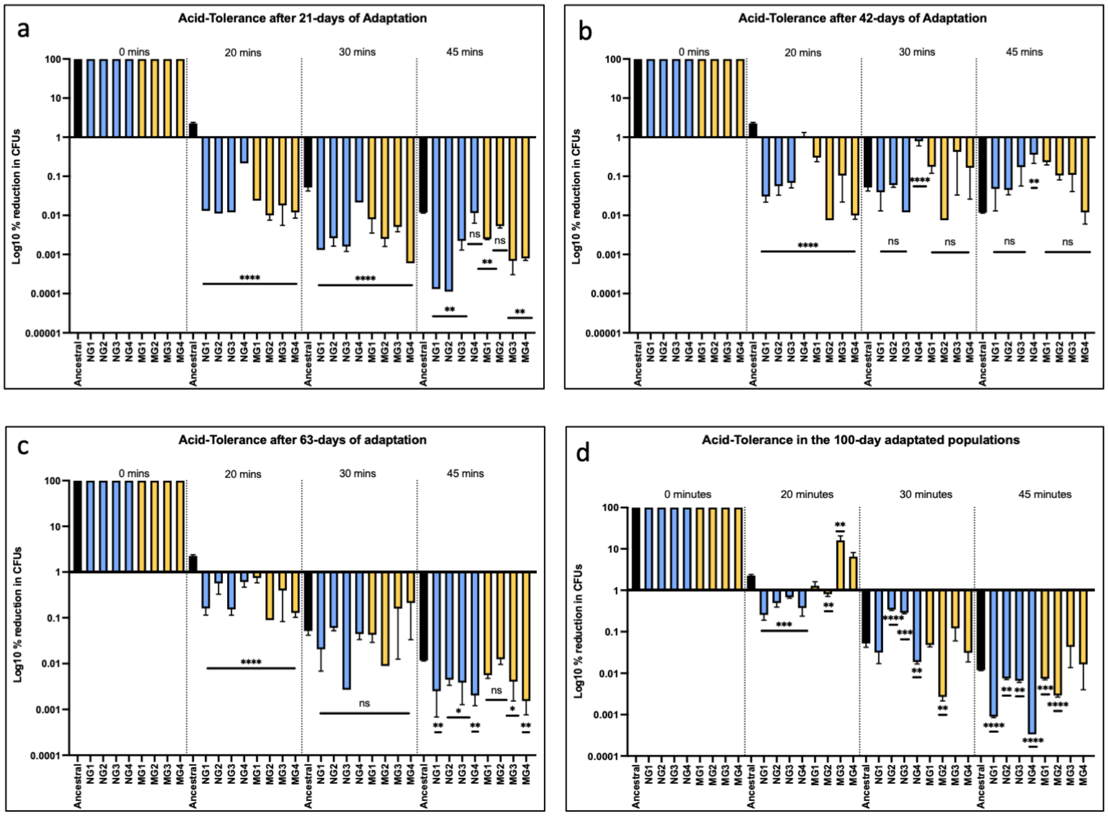

Supplementary Figure 2 – The acid tolerance fluctuates through adaptation. Acid tolerance was assessed after A) 21- B) 42- C) 63- and D) 100-days of adaptation. Data shows that this phenotype is extremely variable not only between the two environments but also between adaptation time and between biological replicates. Data was plotted in GraphPad Prism ® 9.2.0 and unpaired t-tests with 95% confidence were used to calculated significant differences for pairwise comparisons between the ancestral and each treatment populations. Error bars are standard error means and significance from T-tests are reported as a two-tailed p-value where * ≤ 0.05, ** ≤ 0.01, *** ≤ 0.001 and **** ≤ 0.0001.

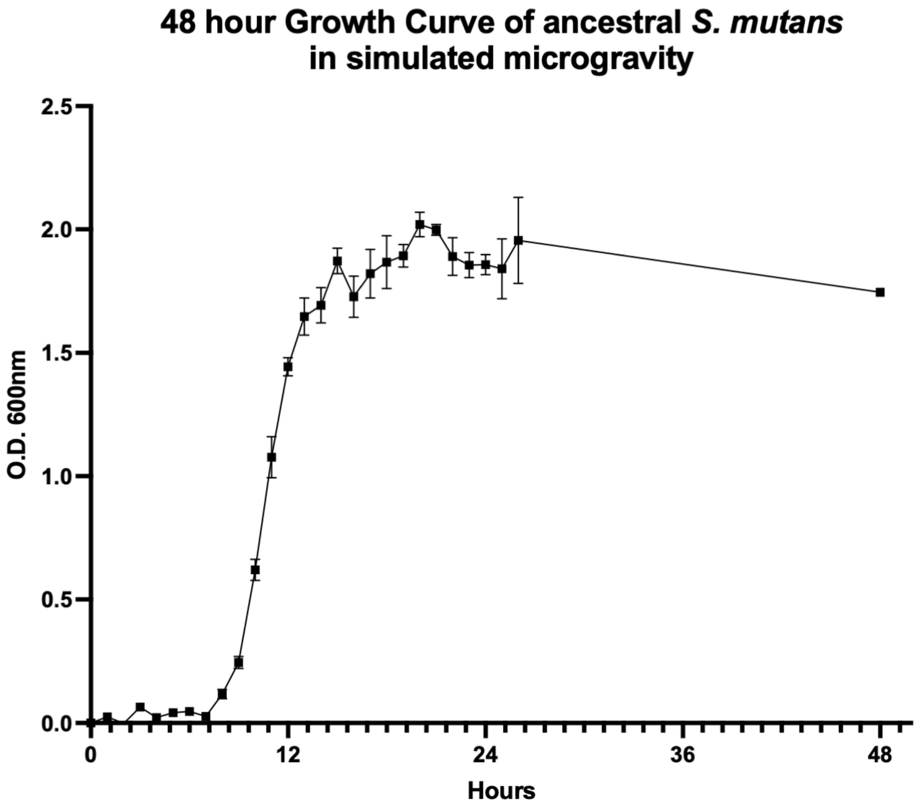

Supplementary Figure 3- 48-hour growth curve. Ancestral *S. mutans* was cultured for 48-hours in BHI media in the HARVs positioned to simulate microgravity. Samples were collected every hour for 24 hours with a final reading at 48-hours. This graph was used to determine an ideal subculturing time for the selection experiment and to estimate generation time. Error bars are standard error mean.

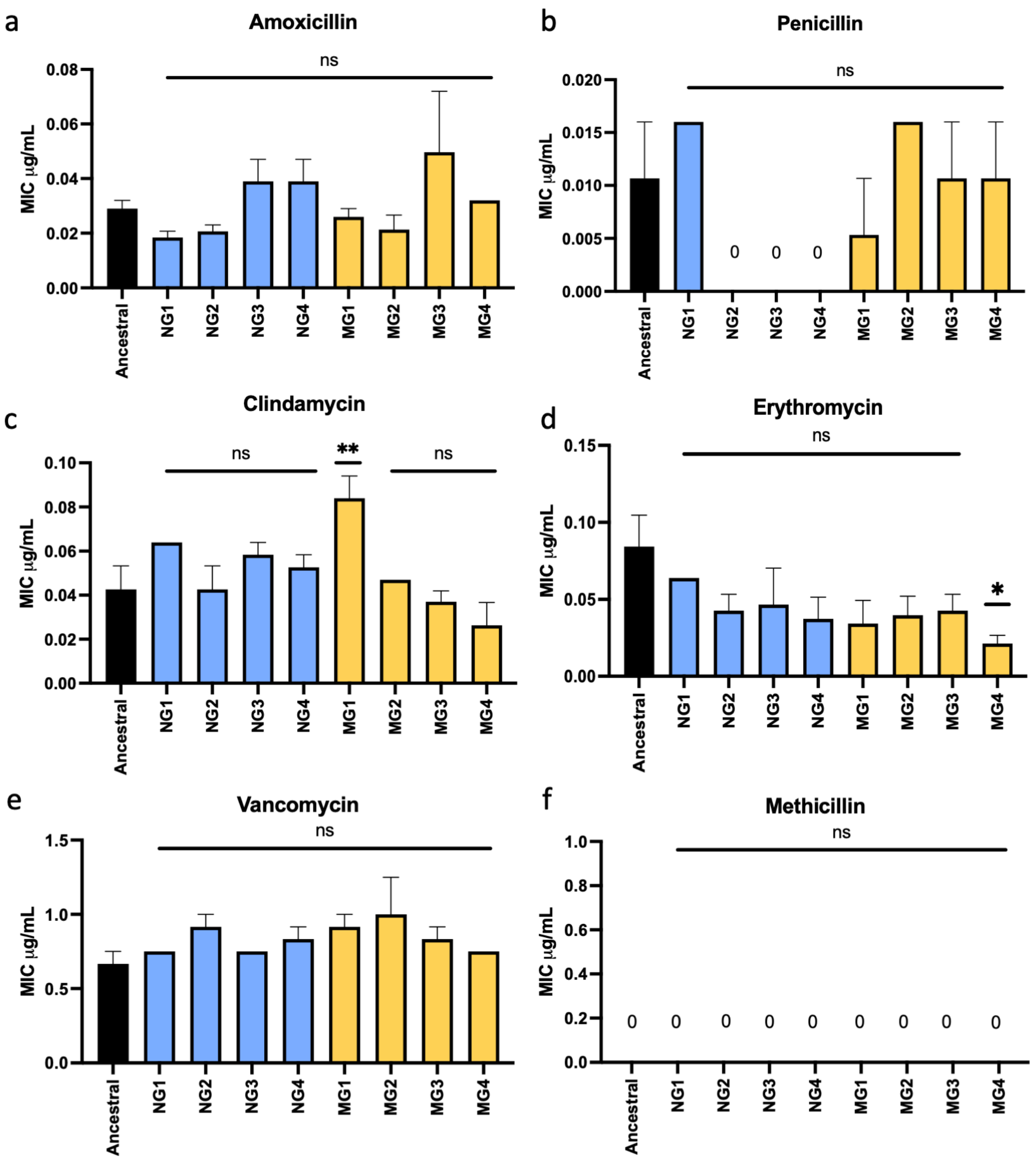

Supplementary Figure 4 – Antibiotic susceptibility using Etest® strips. Antibiotic susceptibility was performed to determine minimum inhibitory concentrations (MIC) towards six traditional antibiotics used to treat dental infections. Each population was assessed three times and MICs were read from the strip and recorded. Error bars are standard error means and significance is reported as a two-tailed p-value from T-tests where * ≤ 0.05, ** ≤ 0.01, *** ≤ 0.001 and **** ≤ 0.0001.
